# Modifiable risk factors of vaccine hesitancy: insights from a mixed methods multiple population study combining machine learning and thematic analysis during the COVID-19 pandemic

**DOI:** 10.1186/s12916-025-03953-y

**Published:** 2025-03-12

**Authors:** Omid V. Ebrahimi, Ella Marie Sandbakken, Sigrun Marie Moss, Sverre Urnes Johnson, Asle Hoffart, Sarah Bauermeister, Ole André Solbakken, Lars T. Westlye, Esten H. Leonardsen

**Affiliations:** 1 Department of Experimental Psychology, University of Oxford, Anna Watts Building, Woodstock Rd, Oxford, OX2 6GG UK; 2 Department of Psychiatry, University of Oxford, Oxford, UK; 3 Department of Psychology, University of Oslo, Oslo, Norway; 4 Modum Bad Psychiatric Hospital and Research Center, Vikersund, Norway; 5 Department of Psychology, Oslo New University College, Oslo, Norway; 6 Centre for Precision Psychiatry, Division of Mental Health and Addiction, Institute of Clinical Medicine, Oslo University Hospital &, University of Oslo, Oslo, Norway; 7 KG Jebsen Centre for Neurodevelopmental Disorders, University of Oslo, Oslo, Norway

**Keywords:** Vaccine hesitancy, Mixed methods study, Machine learning, Extreme Gradient Boosting (XGBoost), Thematic analysis, General adult population, COVID-19, Pandemic

## Abstract

**Background:**

Vaccine hesitancy, the delay in acceptance or reluctance to vaccinate, ranks among the top threats to global health. Identifying modifiable factors contributing to vaccine hesitancy is crucial for developing targeted interventions to increase vaccination uptake.

**Methods:**

This mixed-methods multiple population study utilized gradient boosting machines and thematic analysis to identify modifiable predictors of vaccine hesitancy during the COVID-19 pandemic. Predictors of vaccine hesitancy were investigated in 2926 Norwegian adults (*M*_age_ = 37.91, 79.69% female), before the predictive utility of these variables was investigated in an independent sample of 734 adults in the UK (*M*_age_ = 40.34, 57.08% female). Two independent teams of authors conducted the machine learning and thematic analyses, blind to each other’s analytic procedures and results.

**Results:**

The machine learning model performed well in discerning vaccine hesitant (*n* = 248, 8.48% and *n* = 109, 14.85%, Norway and UK, respectively) from vaccine uptaking individuals (*n* = 2678, 91.52% and *n* = 625, 85.15%), achieving an AUC of 0.94 (AUPRC: 0.72; balanced accuracy: 86%; sensitivity = 0.81; specificity = 0.98) in the Norwegian sample, and an AUC of 0.98 (AUPRC: 0.89; balanced accuracy: 89%; sensitivity = 0.83; specificity = 0.97) in the out-of-sample replication in the UK. The mixed methods investigation identified five categories of modifiable risk tied to vaccine hesitancy, including illusion of invulnerability, doubts about vaccine efficacy, mistrust in official entities, minimization of the societal impact of COVID-19, and health-related fears tied to vaccination. The portrayal of rare incidents across alternative media platforms as fear amplifiers, and the mainstream media’s stigmatizing presentation of unvaccinated individuals, were provided as additional motives underlying vaccine reluctance and polarization. The thematic analysis further revealed information overload, fear of needles, previous negative vaccination experiences, fear of not getting healthcare follow-up after vaccination if needed, and vaccine aversion due to underlying (psychiatric) illness (e.g., eating disorders) as motives underlying vaccine hesitance.

**Conclusions:**

The identified influential predictors were consistent across two European samples, highlighting their generalizability across European populations. These predictors offer insights about modifiable factors that could be adapted by public health campaigns in mitigating misconceptions and fears related to vaccination toward increasing vaccine uptake. Moreover, the results highlight the media’s responsibility, as mediators of the public perception of vaccines, to minimize polarization and provide accurate portrayals of rare vaccine-related incidents, reducing the risk aggravating fear and reactance to vaccination.

**Supplementary Information:**

The online version contains supplementary material available at 10.1186/s12916-025-03953-y.

## Background

Vaccine hesitancy is ranked among the world’s leading health challenges, labeled as one of the top 10 threats to global health by the World Health Organization [1]. This phenomenon is defined as a delay in acceptance or reluctance to vaccinate despite the availability of efficacious vaccines [2, 3]. A key factor promoting vaccine hesitancy as a major global health problem is its ability to reverse progress made in tackling vaccine-preventable disease [1, 4]. Moreover, vaccination is a key proactive measure in protecting against illness, with increases of vaccine coverage estimated to reduce an additional of 1.5 million disease-related deaths per year worldwide [1, 5].

The onset of the recent SARS-CoV-2 pandemic highlighted the general public’s dependency on the availability of efficacious vaccines across the globe, with the course of the pandemic being interconnected with the existence and routine uptake of vaccination [6, 7]. The successful implementation of mass vaccination programs, however, depends on the public’s willingness to vaccinate, rendering vaccine hesitancy as a critical problem during current and forthcoming health crises [7, 8].

Several studies have outlined challenges with vaccine uptake across nations, calling for research to identify predictors of vaccine hesitance [9–11]. Specifically, more knowledge is needed about the psychological mechanisms predicting vaccine hesitancy, with these processes likely to be informative across different contexts [12]. An optimal prediction of vaccine hesitancy would constitute the identification of a limited set of key variables with high predictive power, maximizing applicability and practicality of developing public health measures designed to increase vaccine uptake [13]. Moreover, testing the predictive utility of identified predictors in independent samples is imperative to examine their generalizability across populations [14]. It would further be of added utility to identify modifiable factors that are subjectable to change (e.g., risk perception of vaccines) above more stable risk factors such as lower education levels and biological sex [15].

One promising category of modifiable risk factors are cognitions and attitudes [3]. This includes beliefs about the efficacy of vaccination in mitigating societal transmission rates, perceived danger of the virus for societal functioning, and degree to which individuals believe vaccination can effectively protect them against infection [16]. Unlike stable demographic risk factors, these variables can fluctuate and change within individuals, providing opportunities for intervention. Moreover, cognitive and attitudinal factors can play a key role in shaping health behaviors, rendering them important to investigate [17, 18]. In building models incorporating such modifiable factors, it is important to rely on the existing literature to investigate the comparative utility of novel variables against previously identified predictors of hesitancy, facilitating the identification of variables with the greatest importance in predicting hesitance. Previously, a range of demographic factors have been tied to vaccine hesitancy, including unemployment, lower education, younger age, rural residency, female sex, and migrant status [19, 20]. Additionally, sociopolitical and contextual features previously related to hesitancy include governmental trust, COVID-19 anxiety, perceived risk of infection, and social media use [19, 21].

Accordingly, controlling for the impact of these known risk factors, we leveraged the large-scale Norwegian MAP-19 study [22], collected during the COVID-19 pandemic, to investigate the predictive utility of a range of beliefs and attitudes about vaccination, above and beyond sociodemographic variables previously identified to be related to vaccine hesitancy in the literature. To investigate the generalizability of the findings and the utility of identified key predictors across nations, we aimed to perform an out-of-sample replication in a large-scale sample representative of the UK adult population [23, 24].

While machine learning models are powerful for identifying key factors that predict vaccine hesitancy, this predictive advantage can come at the cost of explanation [25, 26]. Moreover, a general limitation with quantitative analyses includes that the use of pre-defined and standardized measures reduces the opportunity to identify novel processes that may be related to hesitancy in an open and unrestricted manner [27]. Accordingly, to increase insight about cognitions and mechanisms related to vaccine hesitancy during the COVID-19 pandemic, we supplemented these predictive models with a qualitative thematic analysis [28] on all the subjects who provided written open-ended explanations for their hesitance. This mixed methods study design allows a more detailed and nuanced understanding of vaccination hesitance [29]. To avoid influence from one set of analytic results to the other, two groups of researchers conducted each set of analyses (i.e., machine learning and thematic analysis) independently.

In summary, this study sought to identify (a) the most influential modifiable predictors of vaccine hesitancy during the COVID-19 pandemic while controlling for demographic characteristics related to the phenomena; (b) investigate the generalizability of the findings by conducting an out-of-sample replication to examine the utility of identified influential predictors in an independent sample from another nation; in addition to (c) provide a more nuanced and comprehensive understanding of vaccine hesitancy through a qualitative investigation of respondents’ motives underlying their hesitance.

## Methods

The present study is part of The Norwegian COVID-19, Mental Health and Adherence Project (MAP-19), a large longitudinal study investigating mental health and preventive health behaviors in the adult population during the COVID-19 pandemic [22]. The study was approved by The Norwegian Regional Committee for Medical and Health Research Ethics (REK; reference: 125510) and the Norwegian Centre for Research Data (NSD; reference: 802810).

### Study design, participants, and procedure

Eligible participants included all adults (age ≥ 18 years) residing in Norway who provided informed consent to participate in the study. Upon initial recruitment in March 2020 (T1), participants joined through participation in an online survey disseminated to a random selection of Norwegian adults through a Facebook Business algorithm, in addition to systematic dissemination of the survey via national, regional, and local information platforms (i.e., television, radio, and newspapers). This procedure is elaborated in detail elsewhere [30, 31]. The sampling procedure involved the recruitment of a proportional number of subjects from each region of the country with respect to the region’s population size. Data from the eighth wave of the MAP-19 study (T8; collection period: January 2 to January 14, 2022) was used for the present study, where COVID-19 vaccine hesitancy along with beliefs and attitudes about vaccination were measured. In total, 2926 adults provided data at this assessment wave, rendering them eligible for our analyses. Among these, 248 participants (8.48%) were vaccine hesitant, corresponding to the known rate of adults unvaccinated against COVID-19 (approximately 9%) in the Norwegian population around the measurement period [32]. Respondents had the opportunity to receive a pair of noise cancelling headphones for participation. Data quality was further examined through attention checks [33]. This was performed by asking participants the question: “Please provide the response “a little” if you are paying attention to this survey” (response options: not at all; a little, moderately; a lot; extremely). 97.80% of the subjects passed the attention checks, with subjects failing these being excluded from the sample to maintain high data quality.

### Measurement

A list of all variables used by the predictive algorithm in the present study, along with their description and coding, is provided in Additional Document 1. Examples of the categories of variables used are presented below.

#### Vaccine hesitancy

Following assessment practices in the literature, vaccine hesitancy was measured by querying participants about whether they planned to vaccinate against COVID-19 upon availability of a vaccine, with adults responding no coded as hesitant (binary scale: hesitant: 1; willing: 0), yielding the label (i.e., outcome variable) of the study [11].

#### Sociodemographic predictors

The subjects provided sociodemographic information such as their biological sex (females: 0; males: 1), age (continuous in years; age groups: 18–30 years; 31–44 years; 45–64 years; and 65 years and above), relationship status (in a relationship: 1; single: 0), and education level (compulsory school: 0; upper secondary high school: 1; student: 2; any university degree: 3). Participants further provided information about their current living situation, urban versus rural residency, cultural background, and employment status (see Additional File: Document 1 for full details).

#### Medical and psychiatric comorbidities

Information regarding the presence of a preexisting psychiatric comorbidity, medical comorbidity, and presence of a chronic illness was obtained. Mental health covariates were included using validated instruments measuring general anxiety symptoms (GAD-7; [34]), sleep difficulties (BIS; [35]), post-traumatic stress symptoms (PCL-5; [36]), and depression (PHQ-9; [37]).

#### Contextual variables

Participants reported their general level of adherence to pandemic mitigation protocols including social distancing and hygienic behavior recommendations, governmental trust, frequency of information acquisition about the pandemic from different information sources, financial and occupational concerns, worries related to self-infection, and fear about significant others being infected by the SARS-CoV-2 virus [38] (Additional File: Document 1).

#### Cognitions and beliefs related to vaccination and the pandemic

Attitudes and beliefs related to vaccination, the pandemic, and the SARS-CoV-2 virus were measured by asking subjects to rate the extent to which they agreed with different statements, measured on a 5-point Likert scale (1–5; completely disagree: 1 to completely agree: 5). For instance, overconfidence in ability to avoid the SARS-CoV-2 virus was measured through the statement “I do not need to vaccinate as I have managed to avoid the coronavirus so far”. Other measured cognitions and beliefs included fear of vaccination due to side-effects or related to an underlying illness, extent of belief in whether vaccines were developed too fast to be safe, perceived dangerousness of COVID-19 as an overall societal problem, extent of trust in information disseminated about vaccines from health-care officials, extent of belief in the ability of vaccines to effectively mitigate societal transmission rates, protect against COVID-19 infection, and the perceived health risk associated with vaccinating (Additional File: Document 1). The extent to which the subjects believed in the superiority of natural immunity as compared to vaccination was further measured.

### Machine learning analyses

The machine learning analysis was performed by the first and last author, blind to the analytic procedures and results of the qualitative team (second and third authors), and vice versa.

#### Data splitting

To evaluate the performance of our models in an unbiased manner, and obtain a singular list of variable importances, we reserved 1/3 of our dataset for testing (test set, *n*_test_ = 975) before doing any model fitting. The splitting was done while stratifying on vaccine hesitancy and a list of demographic and psychological variables (Additional File: Table S1) to get a similar distribution between the folds (i.e., subsets of data). We used the same strategy to split the remaining 2/3 of the dataset, constituting the training set (*n*_train_ = 1951), into five similar folds to facilitate cross-validation for tuning hyperparameters.

#### Variable selection and encoding

When predicting vaccine hesitancy, we used a set of 111 variables, comprising of mentioned demographic factors and psychological variables such as attitudes and cognitions all measured prior to or at the same time as the outcome (detailed in Additional File: Document 1). Beyond attempting to select variables with minimal overlap to reflect distinct constructs in the variable selection procedure, decision-tree models (i.e., XGboost) handle any potential multicollinearity well [39]. The outcome variable (vaccine hesitancy) was coded as a binary variable (hesitant: 1; willing: 0). For the predictors, missing data was retained and handled as a distinct level by the trained models [40].

#### Model fitting

All analyses were run using Python 3.8.6 on a machine running Windows Server 2012. Gradient boosting machines [41] were fitted using the XGBoost library version 1.6.1 [42]. All models were trained to optimize the log loss:

$$\frac1N\sum\limits_i-\left(y_i\ast\text{log}\left({\widehat y}_i\right)+\left(1-y_i\right)\ast\text{log}\left(1-{\widehat y}_i\right)\right)$$

where ${y}_{i}$ denotes the response by person *i*, ${\widehat{y}}_{i}$ the predicted response for person *i*, and *N* the number of participants. To find optimal hyperparameters, we performed an inner cross-validation in the training set, searching for parameters in a complete grid [43]. Due to the sample size restrictions, we constrained our search to five parameters with either two or three possible values (Additional File: Table S2), resulting in 108 models per fold. For model selection in the cross-validation loop, we used the area under the receiver operating characteristic curve (ROC AUC, AUC for short) [44], selecting the hyperparameter settings that yielded the highest average AUC across folds.

#### Model evaluation

In the final model evaluation, we mainly relied on the AUC. Given the ability of different performance metrics to capture unique properties of the model [45], we computed a set of complementary evaluation metrics, defined below. All metrics rely on the number of true positives (*tp*), true negatives (*tn*), false positives (*fp*), and false negatives (*fn*):

$$Accuracy =\frac{\left(tp + tn\right)}{\left(tp + tn + fp + fn\right)}$$

$$Balanced\ accuracy =\frac{1}{2} \left(\frac{tp}{tp+fn}+\frac{tn}{tn+fp}\right)$$

$$Precision = \frac{tp}{\left(tp + fp\right)}$$

$$Recall\ (Sensitivity) = \frac{tp}{\left(tp + fn\right)}$$

$$Specificity = \frac{tn}{\left(tn + fp\right)}$$

Area under the precision-recall curve (AUPRC) was approximated with the average precision score (AP), calculated as:

$$AP=\sum\limits_n\left(R_n-R_{n-1}\right)P_n$$

where ${R}_{n}$ denote recall and ${P}_{n}$ denote precision at threshold $n$.

The aforementioned metrics are all contingent on a threshold to dichotomize what is classified as hesitant versus willing (the classification threshold), often set to 0.5. In the present study, models were selected using the AUC, which does not rely on accurately setting this classification threshold. Thus, we observed that the threshold used by the best performing model was poorly tuned [46]. To alleviate this, we used the ROC curve from the training data to set a moving classification threshold for dichotomization [47]. This was based on the True Positive Rate, $tpr=\frac{tp}{\left(tp + fn\right)}$, and the False Positive Rate, $fpr=\frac{fp}{\left(fp + tn\right)}$, at each threshold *t* in the ROC curve. From these, a geometric mean was computed:

$${g}_{t}= \sqrt{tp{r}_{t}* \left(1 - fp{r}_{t}\right)}$$

Next, we selected the *t* yielding the highest geometric mean, ${t}^{*}=\underset{t}{\text{max}}{g}_{t}$, which in the training set was empirically determined as ${t}^{*}=0.13$. This value represents the optimal classification threshold and was used in the calculations of the aforementioned metrics in the test set. Substantively, adjusting the classification threshold is useful to achieve a more balanced performance between detecting true positives and avoiding false negatives, a technique which has been found to be well-suited for imbalanced classification problems [48].

#### Variable importance

To determine variable importance, we used the default XGBboost functionality based on calculating the average gain for each split using each predictor. For an individual branching in each decision tree, the gain denotes how much information is obtained by performing the split, measured by how much it decreases the prediction error:

$$Gain =\frac{1}{2}* \left(\frac{{G}_{L}^{2}}{{H}_{L}+ \lambda }+\frac{{G}_{R}^{2}}{{H}_{R}+ \lambda }-\frac{{\left({G}_{L}+ {G}_{R}\right)}^{2}}{{H}_{L}+ {H}_{R}+ \lambda }\right)- \gamma$$

where *G*_*{L,R}*_ and *H*_*{L,R}*_ describe the first and second derivatives of the loss for all samples that end up in the given node of the tree, and *λ* (L2-loss) and *γ* (minimum loss reduction required for further splitting) are regularization terms. To investigate the differences between the willing versus the vaccine hesitant individuals in the most important variables, we performed post hoc inspections of the differences in the distributions of these variables across the two groups.

#### Independent sample replication

For replication, we used a large open-source study from the UK collected during the COVID-19 pandemic using identified variables resembling those in our original dataset [23]. To maximize the overlap of variables in the UK dataset and the main (Norwegian) dataset while retaining a large enough sample, we used the data collected at wave 5 (collection period: March 24 to April 20, 2021) in the UK data. We trained a new model to predict vaccine hesitancy, encoded in the binary target variable *W5_C19_Vax_Self*, asking whether participants would take a vaccine for COVID-19 when it becomes available to them [23]. We selected all variables in this data set which resembled the 10 most important variables in our model trained on the Norwegian dataset, resulting in the selection of 18 variables from the UK data (Fig. 2 and Additional File: Table S3). Also here, we split the data into a test split and a training split (*n*_train_ = 490, *n*_test_ = 244) and performed an inner fivefold cross-validation to find optimal hyperparameters [43]. The hyperparameter settings tested were the same as for the original model (Additional File: Table S2).

### Thematic analysis

Without insight into the quantitative design or analysis of the material, the second and third authors independently analyzed the qualitative survey material. Out of the 248 vaccine hesitant subjects in the Norwegian sample, 53 filled out the open-ended question: “If you did not get vaccinated against COVID-19, could you please explain what contributed to you not wanting to get vaccinated?”. Of the 53 responses, one was removed as it was too limited to analyze (the respondent merely answered “no”). We were left with 52 extracts (range 4 to 206 words, mean response word length 51 words), male 9, $\overline{age }$ 51.8, range 20–70; female 43, $\overline{age }$ 40.3, range 20–84.

We adopted a template analysis approach to thematic analysis [28, 49] with a critical realist epistemological stance [50]. Aiming for transparency, we have incorporated an audit trail (as per for example: [51, 52]) to allow readers access to and overview of the analytic process (see Additional File: Document 2). The second and third author separately coded all the qualitative data material for manifest meaning and latent meaning. Based on this, we developed an initial template in the form of an elaborate, hierarchical coding template (8 superordinate codes, 2–6 subordinate under each, and up to 3 sub-subordinate codes, with a total of 50 codes across those three levels; Additional File: Document 2). This initial coding template was developed inductively to allow for open analysis and combating the risk of “foreclosure of analysis” [53]. Having developed the template, the second and third author separately coded the whole dataset using the template. Of 317 coding instances, 282 coding instances overlapped at main theme level (88.96%) between the authors, and 260 overlapped at sub-theme level (82.02%). Where the authors had coded differently, this was resolved through discussion and refinement of the coding template. Thereafter, and based on the separate coding and the discussions of the coding, we generated the final coding template, presented as a hierarchical thematic map, consisting of six main themes, 2–5 subthemes under each, and 0–6 sub-subthemes (see Fig. 3 for overview). All extracts used in the article are translated from Norwegian and anonymized to maintain confidentiality.

### Open science, transparency, and reproducibility

The machine learning analyses were performed using Python 3.8.6, with our code openly available. The Jupyter Notebook documentation of the full procedure and findings can be found here (https://osf.io/nbrwz/), where also the visualized plots are readily viewable upon opening the code files in a matching coding environment (e.g., Visual Studio Code, Jupyter Notebook). The audit trail of the thematic analysis can be found in Additional File: Document 2, which provides a transparent documentation of the analytical process in a step-by-step manner [52, 54]. The ethical approval for the Norwegian dataset precludes submission of data to public repositories. The UK data is openly available and can be found at the online repository of the Center for Open Science (https://osf.io/v2zur/).

## Results

Among the 2926 participants eligible for the study in the Norwegian sample, ages ranged from 18 to 86 years ($\overline{age }$ = 37.91), with 2332 (79.69%) being female, 179 of ethnic minority status (6.16% non-white Norwegian), and 1767 (60.39%) having a university degree. A total of 248 individuals (8.48%) reported hesitance to vaccinate. The distribution of these demographic variables in the vaccine hesitant group is reported in Additional File: Table S4, and the percentage of missingness among variables with missing values in Additional File: Table S5. The percentage of participants reporting preexisting mental health conditions in this sample was 17.98%, representative of the known rate of psychiatric disorders in the Norwegian adult population, which is between 16.66 and 25.00% [55]. The quota of participants sampled from each region of Norway was further proportional to the respective region’s size [30].

### Predictive performance

**Fig. 1:**
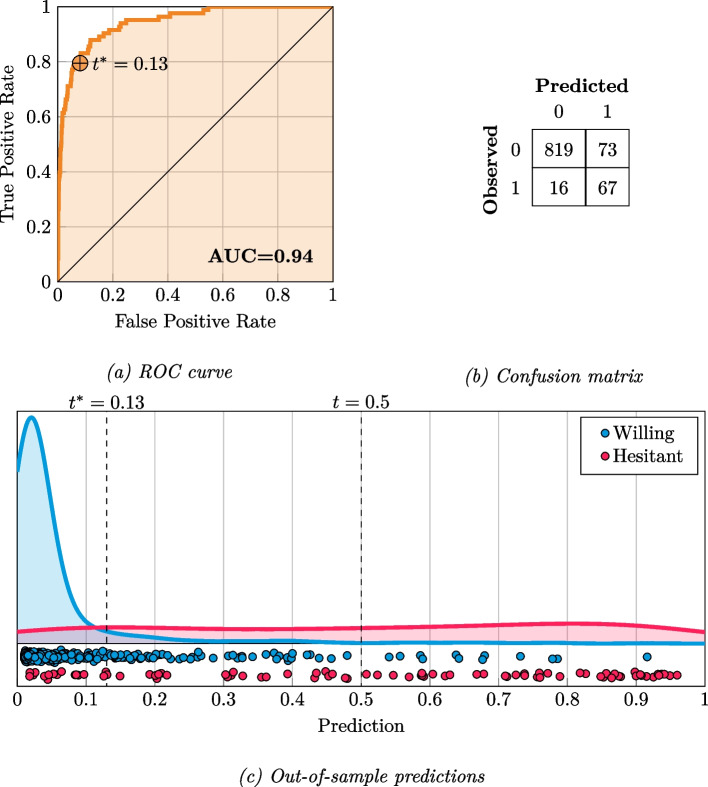
Predictive performance of the best model in the test fold of the Norwegian sample. ROC curve: Receiver Operating Characteristic curve; AUC: Area under the ROC curve; t: Classification threshold

In the independent test set of the Norwegian sample, the best model achieved an AUC of 0.94, corresponding to a balanced accuracy (with an adjusted classification threshold, see Methods) of 86.27% (Table 1 and Fig. 1). The model was able to correctly identify a large portion of those willing to vaccinate (*Specificity* = 0.98) and further a substantial amount of those hesitant (*Sensitivity* = 0.81). Partially due to the class imbalance, the positive predictive value was low (*Precision* = 0.48). The impact of class imbalance on model behavior was further emphasized in a F1-score of 0.63 and an AUPRC of 0.72, presenting more modest results than the AUC in the Norwegian sample. Overall, when using an appropriate classification threshold, the model performed well at discerning vaccine hesitant from willing participants.

**Table 1:** Classification metrics for the best performing model

	Default classification threshold		Optimal classification threshold
		**Norwegian sample**	
AUC		0.94	
AUPRC		0.72	
Brier score		0.04	
F1-score		0.63	
Accuracy	94.77%		90.87%
Balanced accuracy	75.83%		86.27%
Recall (sensitivity)	0.53		0.81
Precision (PPV)	0.79		0.48
Specificity	0.96		0.98
		**UK sample**	
AUC		0.98	
AUPRC		0.89	
Brier score		0.04	
F1-score		0.72	
Accuracy	94.69%		94.28%
Balanced accuracy	89.09%		89.96%
Recall (sensitivity)	0.81		0.83
Precision (PPV)	0.83		0.79
Specificity	0.96		0.97

### Influential predictors

**Fig. 2:**
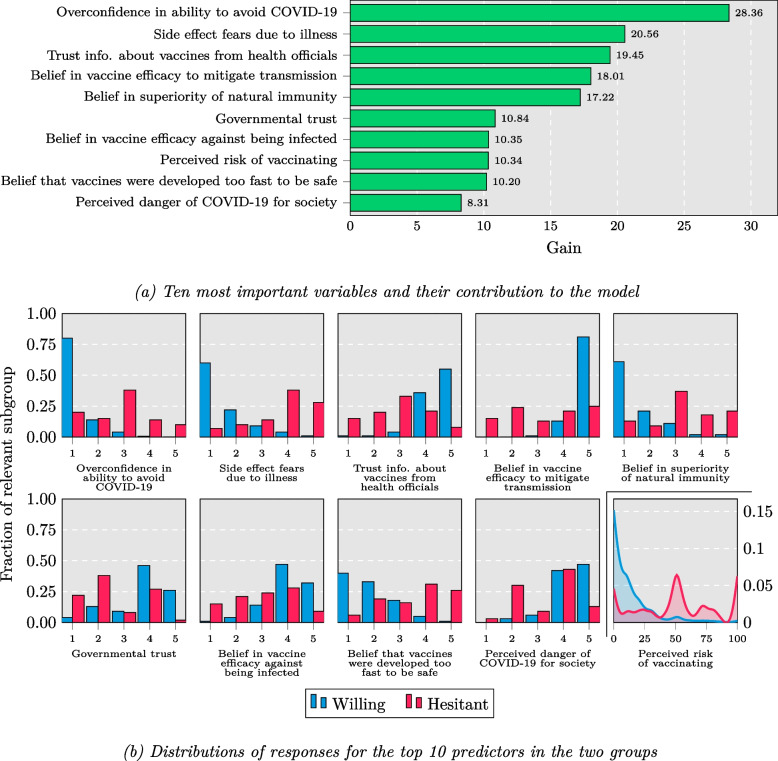
Role of the key predictors in the model predicting vaccine hesitancy in the Norwegian sample. **a** Variable importances were calculated based on information gain in the training set. **b** The distribution for each predictor is visualized based on the hold-out test set. The first nine variables in Figure 2b are ordinal variables, ranging from 0 to 5 (Completely Disagree to Completely Agree for variable 1–3 and 5–8; and Not at all probable to Highly probable for variable 4; and Not at all serious to Very serious for variable 9), whereas variable 10 was continuous, ranging from 0 to 100 (Not risky at all to Maximally risky)

The importance of the individual predictors were assessed based on the resulting information gain from their inclusion in the model (see Methods). The 10 variables most influential for predicting vaccine hesitancy are all listed in Fig. 2a. The variable yielding the highest predictive utility was overconfidence in one’s ability to avoid the COVID-19 virus. Post hoc analyses (Table 2 and Fig. 2b) revealed that the distribution of responses for this variable in the willing subgroup of the test set was narrow (*mode*_willing_ = 1, *SD*_willing_ = 0.59), reflecting that most vaccine willing individuals showed similar extents and displayed low levels of this belief. In the vaccine hesitant group, responses were generally more spread out, with a greater proportion of subjects shifted toward having higher confidence in their ability to avoid the virus (*mode*_hesitant_ = 3, *SD*_hesitant_ = 1.22), as portrayed in Fig. 2b. Examples of other influential predictors of hesitancy included fear of side-effects due to illness, distrust in information about vaccines from health officials, doubts about the efficacy of vaccination in both mitigating transmission in society and protecting against infection, believing in the superiority of natural immunity over vaccination, that vaccines were developed too fast to be safe, governmental distrust, and minimization of COVID-19 as a societal problem.

**Table 2:** Summary of the distribution of the responses for the 10 most important variables for the vaccine hesitant and willing group in the independent test set of the Norwegian sample

	**Willing**			**Hesitant**		
**Variable**	***mode***	***mean***	***SD***	***mode***	***mean***	***SD***
Overconfidence in ability to avoid COVID-19	1	1.26	0.59	3	2.80	1.22
Side effect fears due to illness	1	1.64	0.96	4	3.71	1.19
Trust information about vaccines from health officials	5	4.45	0.74	3	2.87	1.16
Belief in vaccine efficacy to mitigate transmission	5	4.75	0.62	5	3.17	1.43
Belief in superiority of natural immunity	1	1.61	0.94	3	3.25	1.26
Governmental trust	4	3.78	1.10	2	2.48	1.18
Belief in vaccine efficacy against being infected	4	4.05	0.86	4	2.95	1.23
Perceived risk of vaccinating	0	14.00	17	50	48.00	29
Belief that vaccines were developed too fast to be safe	1	1.95	0.98	4	3.53	1.23
Perceived danger of COVID-19 for society	5	4.33	0.77	4	3.33	1.14

Notably, all 10 key predictors of vaccine hesitancy were cognition-related and attitudinal variables, and a similar patterns of differences in response distributions were identified for the 9 other key predictors (Fig. 2a, b) as for the avoidance overconfidence variable, revealing more similar beliefs held in the willing group (typically toward one extreme of the scale) versus more scattered belief patterns in the hesitant group (often with a shifted mode toward either the center or the opposite extreme).

### Independent sample replication results

To further validate our findings, we replicated our predictive analysis in a representative sample from the UK which included a similar set of variables (*n* = 734, $\overline{age }$ = 40.34, *n*_female_ = 419 (57.08%), 14.47% being of non-white British or Irish ethnicity, 26.98% with an undergraduate university degree or higher, 18.12% reporting a pre-existing treatment history with mental health problems). A total of 109 of the 734 participants (14.85%) reported hesitance to vaccinate. The distribution of demographic variables within the vaccine hesitant group can be found in Additional File: Table S4. We selected 18 variables which most closely resembled the top 10 influential variables identified in the predictive model of Norwegian population (Additional File: Table S3), and fit new models using an equivalent data splitting strategy (*n*_train_ = 489, *n*_test_ = 245) and inner cross-validation procedure. Here, the best performing model achieved an out-of-sample AUC of 0.98, AUPRC of 0.89, F-score of 0.72, and a balanced accuracy of 89.96% (Table 1), outperforming the model trained on Norwegian data. In general, beyond the availability of continuous versus ordinal predictors yielding greater statistical power, the differences between hesitant and willing subgroups were more clearly pronounced in the distributions of the predictors in UK versus the Norwegian sample (Additional File: Fig. S1b), yielding an improved prediction. That is, greater polarization in the vaccine-related cognitions and attitudes between the hesitant and willing groups were observed in the UK versus Norway. Compared to the Norwegian model, the UK model had a similarly high specificity (0.98 vs 0.97, Norwegian vs UK sample, respectively) and recall (sensitivity; 0.81 vs 0.83), but significantly higher precision (0.48 vs 0.79). Overall, these results revealed that the predictors identified in the Norwegian population generalize to the UK population.

### Thematic analysis results

**Fig. 3:**
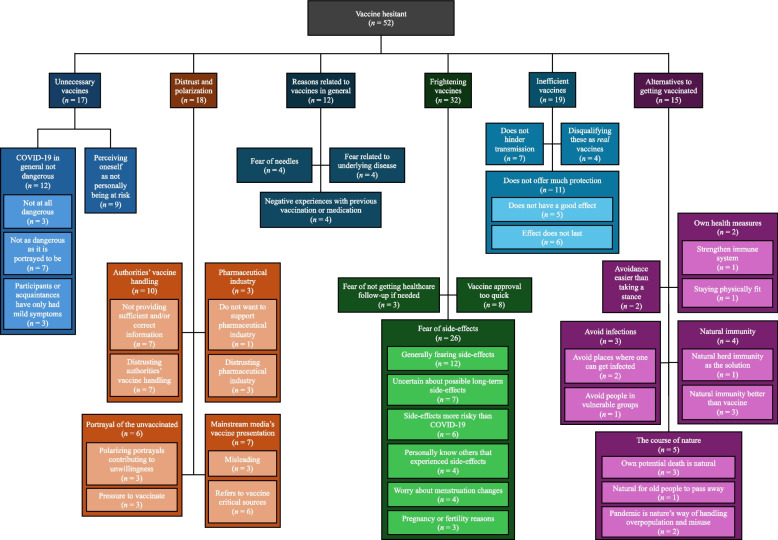
Results of the thematic analysis. The thematic analysis including the main themes and two levels of sub-themes identified through analysis of the participants who also provided open-ended (*n* = 52) explanations underlying their vaccine hesitance

To get more in-depth insight into the participants’ underlying reasons and own explanations of their COVID-19 vaccine hesitancy, the open-ended survey responses (*n* = 52) available in the Norwegian sample were analyzed using a template analysis approach to thematic analysis, resulting in six main themes. Respondents often provided multiple rather than a single reason underlying their vaccine hesitance (an overview of the responses with participant numbers is available in Additional File: Document 2). Figure 3 presents the full thematic map including the main themes and both levels of subthemes, with Table 3 embodying the main themes and the first subtheme level including illustrative quotes from the respondents as characteristic examples for each subtheme.

**Table 3:** Thematic map including the identified main and submotives underlying vaccine hesitancy

Theme	Subtheme	Example extract	*n*
Theme 1: Unnecessary vaccines (*n* = 17)	1.1 Covid-19 in general not dangerous	«(…) Just the concept of walking around being afraid of a cold/flu is absurd» (#35, Woman, 34 years)	12
	1.2 Perceiving oneself as not personally being at risk	«The recommendations for taking the vaccine are based on the average Norwegian. I am younger than the average and much more fit (…)» (#48, woman, 33 years)	9
Theme 2: Inefficient vaccines (*n* = 19)	2.1 Does not offer much protection	«It doesn’t seem like the vaccine has such a good effect, and mutations haven’t been taken into account, as evidenced by the new lockdown.» (#1, man, 20 years)	11
	2.2 Does not hinder transmission	«A vaccine that doesn’t help against transmission, it’s too early to say anything about side-effects.» (#14, woman, 45 years)	7
	2.3 Disqualifying these as *real* vaccines	«These are NOT vaccines. A vaccine gives immunity and hinders transferal. What is being injected into people does neither and it is misleading people to call it vaccine.» (#13, woman, 50 years)	4
Theme 3: Frightening vaccines (*n* = 32)	3.1 Fear of side-effects	«I’m skeptical about long-term side-effects that may arise.» (#36, woman, 33 years)	26
	3.2 Fear of not getting healthcare follow-up if needed	«I don’t have confidence that I’ll receive help from the healthcare system if I experience vaccine side-effects, and I consider it likely that I will get side-effects.» (#43, woman, 26 years)	3
	3.3 Vaccine approvals too quick	«I’m skeptical to that the vaccine had not been tested but was approved without further evaluation.» (#9, woman, 45 years)	8
Theme 4: Distrust and polarization (*n* = 18)	4.1 Authorities’ vaccine handling	«I’m losing trust in everything politicians say because I know they withhold information when looking at what’s happening abroad.» (#31, woman, 40 years)	10
	4.2 Pharmaceutical industry	« (…) It’s especially bad that this is one of the most lucrative things for pharmaceutical companies.» (#38, woman, 32 years)	3
	4.3 Mainstream media’s vaccine presentation	«I read other sources than VG and Dagbladet [mainstream media] and see how many injuries this so-called ‘vaccine’ is causing people.” (#5) (woman, 22 years)	7
	4.4 Portrayal of the unvaccinated	«Public shaming of the unvaccinated leads to a greater degree of reluctance.» (#40, man, 64 years)	6
Theme 5: Alternatives to getting vaccinated (*n* = 15)	5.1 Natural immunity	«Natural immunity has always been the only realistic way out of the pandemic phase.» (#4, woman, 46 years)	4
	5.2 The course of nature	«In the animal world, disease and infection occur when there are too many in the herd, and nature’s way is to sort it out on its own (…). If it’s meant that my time has come, it will come.» (#8, man, 64 years)	5
	5.3 Own health measures	«I want to belong to the control group, emphasizing a strong immune system (vitamin D, vitamin C, and zinc).» (#40, man, 64 years)	2
	5.4 Avoid infections	«I easily have the opportunity to choose or avoid places where the infection is highest.» (#44) (Woman, 66 years)	3
	5.5 Avoidance as easier than taking a stance	«I can’t deal with all the ‘nagging’; the more ‘hassle’ there is around me, the less I can handle it. So for me, it’s easiest (least psychologically painful) to just do nothing.» (#2, woman, 32 years)	2
Theme 6: Reasons related to vaccines in general (*n* = 12)	6.1 Fear of needles	«I have a fear of needles and don’t dare to get vaccinated.» (#6, woman, 36 years)	4
	6.2 Aversion related to own underlying disease	«It’s related to eating disorder issues. I can’t handle putting something into my body that I can’t control.» (#34, woman, years anonymized)	4
	6.3 Negative experiences with previous vaccination	«I have experienced poor health after the swine flu vaccine, I’m afraid, and don’t want to risk worse health due to another vaccine.» (#50, woman, years anonymized)	4

Illustrative quotes from the vaccine hesitant individuals are included for each subtheme

Theme 1, *Unnecessary vaccines* (*n* = 17, prevalence of explanation: 32.69% of the qualitative vaccine hesitant sample), consisted of statements where respondents did not view the vaccines as necessary, either since they did not perceive COVID-19 as dangerous or did not see themselves as being in the risk group. Theme 2, *Inefficient vaccines* (*n* = 19, explanation prevalence: 36.54%), concerned respondents’ views of the vaccines as not offering much protection or mitigating transmission, as well as beliefs disqualifying the vaccines as real vaccines. Theme 3, *Frightening vaccines* (*n* = 32, 61.54%), was the most prevalent motive provided, where fear of the side-effects of the vaccines were emphasized, including worry about both potential short-term and long-term side-effects, fear of not getting healthcare follow-up if needed after vaccination, and worry about the approval of the vaccines as being too quick to be safe. Theme 4, *Distrust and polarization* (*n* = 18, 34.62%), characterized participants who displayed distrust in the authorities’ vaccine handling, in the pharmaceutical industry, or the media’s portrayal of the vaccines. Here, some respondents mentioned that the polarizing and stigmatizing portrayals of the unvaccinated was also a reason for becoming more reluctant to get vaccinated. Theme 5, *Alternatives to getting vaccinated* (*n* = 15, 28.85%), included the beliefs and measures participants listed in handling COVID-19 instead of getting vaccinated, including perceiving natural immunity as a better alternative than vaccination, seeing potential deaths from COVID-19 as the course of nature, taking own health measures such as vitamin supplements, avoiding potential sites of infection such as crowded places, as well as getting overwhelmed with the choice of vaccination and the information overload surrounding the topic and thus mentioning that it was easier to not take a stance about vaccination. Finally, theme 6, *Reasons related to vaccines in general* (*n* = 12, 23.08%), included a general fear of needles, aversion of vaccination related to an existing disease such as eating disorders, and negative experiences with previous vaccinations. Combined, these results map out different underlying motives that were provided related to why hesitant adults did not wish to get vaccinated.

## Discussion

Using two independent analytical teams and datasets across different countries during the COVID-19 pandemic [23, 30], this study aimed to identify a core set of modifiable predictors of vaccine hesitancy in comparison with other variables previously found to be related to this phenomenon. Extending beyond traditional limitations of the predictive paradigm, we further conducted independent qualitative investigations aimed at understanding hesitant individuals’ articulated motives underlying their vaccine hesitance.

Among the 111 variables investigated, our predictive analyses in a sample of Norwegian adults revealed that an accurate prediction of vaccine hesitancy can be achieved through 10 key factors. In line with calls in the literature underscoring the importance of investigating the utility of identified predictors across independent samples [14], we replicated this high prediction accuracy using a comparable set of variables in an independent sample from the UK [23]. This supports the robustness and generalizability of the findings across European populations and corroborates the utility of the identified variables as influential predictors of vaccine hesitance.

Overall, our mixed methods investigation identified five common domains of modifiable risk factors and psychological processes tied to vaccine hesitancy that overlapped across the predictive and qualitative approach.

The first domain concerned an illusion of invulnerability, where overconfidence in one’s ability to avoid the COVID-19 virus and belief in the superiority of natural immunity above vaccination were identified as prominent predictors of hesitancy. This is consistent with a recent study identifying a desire to develop natural immunity and perceiving not needing vaccination as key predictors of hesitancy [56]. The thematic analysis further identified beliefs of not being in a risk group as a motive for not getting vaccinated. This identified underestimation of the risks the SARS-CoV-2 virus suggests a need for targeted communication strategies, which could address these misconceptions by emphasizing the unpredictable nature of novel emergent viruses and the potential for severe outcomes, even for healthy individuals [57, 58]. We also identified hesitant adults reporting that they had taken their own alternative health measures against vaccination, including uptake of vitamin supplements as an alternative to vaccination. This finding highlights the need for evidence-based health literacy campaigns to dispel misconceptions, ensuring that individuals are equipped with accurate knowledge about effective preventive measures and the limitations of alternative health practices in conferring immunity against the virus [59].

The second domain of influential processes concerned health-related fears tied to vaccination, with stronger fear of side-effects, greater perceived risk of vaccination, and believing that the vaccines were developed too fast to be safe [57] predicting hesitancy. This underscores the need for public health measures communicating the rigorous systematic procedures underlying vaccine development, even when expedited [60]. Perceiving the vaccines as potentially harmful was the most frequently listed motive underlying hesitance among the participants. Our open-ended analyses further extend the literature by revealing that some participants were unwilling to vaccinate due to fears of not getting healthcare follow-up if needed after vaccination. This finding reflects a need for communication about the presence of post-vaccination support systems to address and alleviate such concerns, with the potential of fostering a greater sense of safety to mitigate these health-related fears.

The third influential domain of factors underlying hesitance concerned doubts about the efficacy of vaccination, including doubting vaccines’ ability to protect individuals against infection, and the efficacy of the vaccine in mitigating societal transmission overall [61, 62]. Given the evidence of the significant role that vaccination plays in reducing diseases and deaths worldwide (e.g., [63, 64]), this finding highlights the gap between scientific evidence and public perception of vaccinations across certain subgroups of the population. Future studies would do well to combine strategies both aimed at identifying these subgroups and tailored ways to minimize this gap in these respective groups of individuals toward increasing vaccine uptake.

Fourth, degree of trust in official entities was identified as an important predictor of hesitancy. Here, both lower levels of governmental trust in general, and less trust in the information provided by health-care officials about vaccination specifically, predicted vaccination hesitance, highlighting the important role of systemic trust in fostering large-scale behavior change [65, 66]. One strategy that has previously been identified as effective in building trust includes engaging with subgroups through community leaders [67–69], in addition to setting up arenas of dialog allowing for open discussion and addressal of raised concerns [68].

The fifth domain of psychological processes strongly tied to vaccine hesitancy concerned minimization and denial of COVID-19 as a societal problem [62, 70]. While this highlights a general need to combat pandemic-related myths, a recent randomized controlled trial identified that information-based interventions emphasizing personal benefits of vaccination had a greater effect in changing hesitance than information provision pertaining to the societal level, such as the collective benefit of vaccination [71]. Combined with the identified predictors of hesitancy in the present study, these results imply that directing public health information toward individually oriented dimensions such as the illusion of invulnerability against the virus, ways to address health-related fears tied to vaccination, and doubts concerning vaccination efficacy to protect oneself against infection, may prove most efficient in modifying hesitance and increasing vaccine uptake.

Beyond these predictors, a range of novel processes underlying vaccine hesitancy during the COVID-19 pandemic were identified through the analysis of open-ended responses. Several adults noted the media’s impact on their vaccine hesitancy, pointing at cases presented in alternative media sources portraying individuals developing illnesses following vaccination. Given the known cognitive biases tied to vaccine hesitancy which can easily be amplified by media presentations, including availability bias where more vivid and emotionally memorable events are attributed higher weight and are more easily recalled [72], this finding highlights the critical role and responsibility of media outlets as mediators of the public’s perception of vaccination. Moreover, important nuances emerged for the unwillingness to vaccinate among certain individuals, including a general fear of needles, negative experiences with previous vaccinations, and aversions to vaccination related to underlying illness (e.g., eating disorder). This finding suggests the importance of screening for underlying psychiatric conditions and fears which may relate to hesitance, including fear of needles, with a range of effective psychological interventions available for such phobias (e.g., [73, 74]).

The open-ended analysis also identified a hesitant subgroup of adults perceiving disease, infection, and death as part of “the course of nature.” Moreover, adults reported being overwhelmed by the hassle and overload of information around vaccination as a reason for their hesitance, highlighting the importance of identifying the appropriate dosage of information delivery in public health interventions [75, 76]. Notably, some individuals reported polarizing and stigmatizing portrayals of unvaccinated individuals as a key reason for becoming increasingly unwilling to get vaccinated. These findings highlight how stigmatizing portrayals can create an unfavorable “us vs. them” mentality, alienating unvaccinated individuals and making them more resistant to vaccination campaigns, underscoring the necessity for the media, governments, and healthcare communicators to avoid polarizing presentations [77].

While the responses of vaccine hesitant individuals on the identified predictors were tilted toward the opposite extreme compared to the responses of willing individuals, these responses were generally more spread out, indicating more heterogenous attitudes and beliefs in the hesitant compared to vaccine willing individuals. This highlights that these variables perform better in predicting willingness to vaccinate compared to hesitancy, highlighting the need for more research toward identifying additional predictors more strongly predictive of hesitance.

Notably, this study investigated factors related to vaccine hesitancy in the context of the COVID-19 pandemic and in relation to vaccines developed against SARS-CoV-2. There is evidence to support that many of the identified risk factors, including trust in governments and health authorities [78], safety concerns, fear of side-effects, and perceived risk of vaccination [72], extend across different vaccines and diseases [72, 78]. The extent to which other risk factors identified in this study can extend across different vaccines warrants further scrutiny and must be investigated in future studies.

### Strengths and limitations

The present study includes several strengths, including its mixed methods approach using two independent set of researchers who were blind to the investigation procedures and analytical results of one-another, the large-scale investigation of a topic of concurrent and future relevance, and replication of the results in a large independent sample from another nation. This replication and the prediction accuracy across independent samples further corroborates the identification of central mechanisms related to vaccine hesitancy pertaining to the adult population. A notable strength of this study includes that the identified processes related to hesitancy were all factors that are loanable to manipulation by public health interventions. Unlike demographic risk factors such as one’s age group, these modifiable risk factors and processes can be actively modified through public health campaigns, providing key actionable steps for governments and health officials on the route to combating this health-deteriorating global problem. Moreover, in addition to open documentation and code of the full workflow for the machine learning model, we adhered to open science and transparency recommendations in qualitative research [54] by including an audit trail of the thematic analysis to document the analytical process in a step-by-step manner.

This study also includes several limitations. First, the recruitment procedure could have led to self-selection of specific subgroups above others, such as older adults with greater computer use experience. Additional efforts were undertaken to reduce this bias by also recruiting through platforms more accessible through the elderly population. The Norwegian sample was skewed toward oversampling of females and individuals with higher education. Our results were nonetheless replicated in an independent representative sample from the UK, highlighting the limited impact of this skewed sample on our results. Our out-of-sample replication only provides an indirect external validation of the identified predictors, as partial differences between variables across the datasets resulted in the training of a new model in the UK sample using insights from the Norwegian sample, rather than directly testing the Norwegian model on the UK data. This highlights another limitation of this study, with a more direct validation warranted in future research efforts using datasets with fully comparable questionnaires. While adjusting the classification threshold obtained via the ROC curve has been identified as a suitable solution for addressing class imbalance [48], other methods, such as those relying on the Precision-Recall curve, could have been applied. Nonetheless, the identification of influential predictors and selection of the best performing model was based on the AUC, which is unrelated to classification threshold adjustment. As we do not know the extent to which participants’ expressed vaccine hesitancy translated to their real-world vaccination behavior, the sole use of self-report measures is another limitation of this study, calling for future studies based on electronic health records and objective vaccination records. While the open-ended question enabled triangulation and supplementing the machine learning analysis with additional insights about vaccine hesitancy, in-depth interviews would have provided greater nuance and detail about individuals’ motives underlying their hesitancy. Finally, while we investigated out-of-sample replication in an independent sample from another European country, the extent to which these results can be replicated in non-Western cultures and low- and middle-income countries remains to be investigated [79].

## Conclusions

This study identified modifiable psychological processes underlying vaccine hesitance during the COVID-19 pandemic, which could function as targets in evidence-based health literacy campaigns aiming to address misconceptions and fears related to vaccination toward increasing vaccine uptake. Investigating adults across Norway and the UK, we identified five domains of psychological processes predicting vaccine hesitancy, including illusion of invulnerability, vaccine efficacy doubts, mistrust in health officials, minimization of COVID-19’s societal significance, and vaccine-related health fears. Additionally, the portrayal of rare cases amplifying fear across alternative media sources, in addition to stigmatization of unvaccinated individuals in the mainstream media, were highlighted as other underlying motives leading to vaccine reluctance and polarization. These findings, consistent across two independent European samples, underscore the importance of addressing these modifiable factors in targeted public health campaigns to mitigate vaccine hesitance.

## Supplementary Information

Additional File 1: Fig. S1. Role of the key predictors for predicting vaccine hesitancy in the UK sample. (a) The variable importances were calculated based on information gain in the training set. (b) The distribution for each predictor is visualized based on the hold-out test set. All top ten predictors were continuous, ranging from 0 to 100 (Completely disagree to Completely agree for variables 1-2, 6 and 9-10; Not at all to Completely for variables 3-5 and 7; and Not at all threatened to Extremely threatened for variable 8).

Additional File 2: Table S1. Variables used in the data splitting procedure. Table S2: Hyperparameters and the values tested during tuning. The highlighted values are those that produced the best performing model. Table S3: The variables used in the independent sample replication in the UK dataset along with their corresponding variables in the Norwegian sample. Table S4: Proportion of vaccine hesitant individuals (*n* = 248 in Norway and *n* = 109 in UK) within different demographic subgroups. Table S5: Percentage of missingness among the variables with missing values across the vaccine hesitant and vaccine willing subgroups.

Additional File 3. Document 1. List of variables used in the machine learning model.

Additional File 4. Document 2. Audit trail of the analytic procedure in the thematic analysis.

## Data Availability

The UK data supporting the conclusions of this article is openly available and can be found at the online repository of the Center for Open Science (10.17605/OSF.IO/V2ZUR), entitled ‘COVID-19 Psychological Research Consortium (C19PRC) Panel Study’ by McBride and colleagues. For the Norwegian data (MAP-19), the received ethical approval from the Norwegian Centre for Research Data (NSD) precludes submission of raw data to public repositories. Access to the data can be granted from the principal investigator Omid V. Ebrahimi following ethical approval of a suggested project plan for the use of data granted by NSD and REK.

## Abbreviations

AUPRC: Area under the precision-recall curve
MAP-19: Mental Health and Adherence Project
NSD: Norwegian Centre for Research Data
REK: Regional Committee for Medical and Health Research Ethics
ROC AUC: Area under the receiver operating characteristic curve
SARS-CoV-2: Severe acute respiratory syndrome coronavirus 2
XGBoost: Extreme Gradient Boosting

## References

[1] WHO. Ten threats to global health in 2019. Available from: https://www.who.int/news-room/spotlight/ten-threats-to-global-health-in-2019.

[2] Dubé È, Ward JK, Verger P, MacDonald NE. Vaccine hesitancy, acceptance, and anti-vaccination: trends and future prospects for public health. Annu Rev Public Health. 2021;42:175–91. 33798403 10.1146/annurev-publhealth-090419-102240

[3] MacDonald NE. Vaccine hesitancy: definition, scope and determinants. Vaccine. 2015;33:4161–4. 25896383 10.1016/j.vaccine.2015.04.036

[4] Obregon R, Mosquera M, Tomsa S, Chitnis K. Vaccine hesitancy and demand for immunization in Eastern Europe and Central Asia: implications for the region and beyond. J Health Commun. 2020;25:808–15. 33719888 10.1080/10810730.2021.1879366

[5] Ryan J, Malinga T. Interventions for vaccine hesitancy. Curr Opin Immunol. 2021;71:89–91. 34271335 10.1016/j.coi.2021.05.003

[6] Anderson RM, Heesterbeek H, Klinkenberg D, Hollingsworth TD. How will country-based mitigation measures influence the course of the COVID-19 epidemic? The Lancet. 2020;395:931–4. 10.1016/S0140-6736(20)30567-5 PMC7158572 32164834

[7] Sachs JD, Karim SSA, Aknin L, Allen J, Brosbøl K, Colombo F, et al. The lancet commission on lessons for the future from the COVID-19 pandemic. The Lancet. 2022;400:1224–80. 10.1016/S0140-6736(22)01585-9 PMC9539542 36115368

[8] Wiysonge CS, Ndwandwe D, Ryan J, Jaca A, Batouré O, Anya BPM, et al. Vaccine hesitancy in the era of COVID-19: could lessons from the past help in divining the future? Hum Vaccines Immunother. 2022;18:1–3. 10.1080/21645515.2021.1893062 PMC8920215 33684019

[9] Anderson RM, Vegvari C, Truscott J, Collyer BS. Challenges in creating herd immunity to SARS-CoV-2 infection by mass vaccination. The Lancet. 2020;396:1614–6. 10.1016/S0140-6736(20)32318-7 PMC7836302 33159850

[10] Lazarus JV, Ratzan SC, Palayew A, Gostin LO, Larson HJ, Rabin K, et al. A global survey of potential acceptance of a COVID-19 vaccine. Nat Med. 2021;27:225–8. 33082575 10.1038/s41591-020-1124-9 PMC7573523

[11] Neumann-Böhme S, Varghese NE, Sabat I, Barros PP, Brouwer W, van Exel J, et al. Once we have it, will we use it? A European survey on willingness to be vaccinated against COVID-19. Eur J Health Econ. 2020;21:977–82. 32591957 10.1007/s10198-020-01208-6 PMC7317261

[12] Trogen B, Pirofski LA. Understanding vaccine hesitancy in COVID-19. Med. 2021;2:498–501. 33851144 10.1016/j.medj.2021.04.002 PMC8030992

[13] Chowdhury MZI, Turin TC. Variable selection strategies and its importance in clinical prediction modelling. Fam Med Community Health. 2020;8: e000262. 32148735 10.1136/fmch-2019-000262 PMC7032893

[14] Obermeyer Z, Emanuel EJ. Predicting the Future — big data, machine learning, and clinical medicine. N Engl J Med. 2016;375:1216–9. 27682033 10.1056/NEJMp1606181 PMC5070532

[15] Khairat S, Zou B, Adler-Milstein J. Factors and reasons associated with low COVID-19 vaccine uptake among highly hesitant communities in the US. Am J Infect Control. 2022;50:262–7. 34995722 10.1016/j.ajic.2021.12.013 PMC8730806

[16] Davis CJ, Golding M, McKay R. Efficacy information influences intention to take COVID-19 vaccine. Br J Health Psychol. 2022;27:300–19. 34250684 10.1111/bjhp.12546 PMC8420419

[17] Conner M, McEachan R, Taylor N, O’Hara J, Lawton R. Role of affective attitudes and anticipated affective reactions in predicting health behaviors. Health Psychol. 2015;34:642–52. 25222083 10.1037/hea0000143

[18] McEachan R, Taylor N, Harrison R, Lawton R, Gardner P, Conner M. Meta-analysis of the Reasoned Action Approach (RAA) to understanding health behaviors. Ann Behav Med. 2016;50:592–612. 27169555 10.1007/s12160-016-9798-4 PMC4933736

[19] Lincoln TM, Schlier B, Strakeljahn F, Gaudiano BA, So SH, Kingston J, et al. Taking a machine learning approach to optimize prediction of vaccine hesitancy in high income countries. Sci Rep. 2022;12:2055. 35136120 10.1038/s41598-022-05915-3 PMC8827083

[20] Salali GD, Uysal MS. COVID-19 vaccine hesitancy is associated with beliefs on the origin of the novel coronavirus in the UK and Turkey. Psychol Med. 2022;52:3750–2. 10.1017/S0033291720004067 PMC7609204 33070804

[21] Shen AK. Finding a way to address a wicked problem: vaccines, vaccination, and a shared understanding. Hum Vaccines Immunother. 2020;16:1030–3. 10.1080/21645515.2019.1695458 PMC7227626 31799892

[22] Ebrahimi OV, Bauer DJ, Hoffart A, Johnson SU. A critical period for pandemic adaptation: the evolution of depressive symptomatology in a representative sample of adults across a 17-month period during COVID-19. J Psychopathol Clin Sci. 2022;131:881–94. 36326629 10.1037/abn0000786

[23] McBride O, Butter S, Hartman T, Murphy J, Hyland P, Shevlin M, et al. Sharing data to better understand one of the world’s most significant shared experiences: data resource profile of the longitudinal COVID-19 psychological research consortium (C19PRC) study. Int J Popul Data Sci. 2020;5. Available from: https://ijpds.org/article/view/1704. 10.23889/ijpds.v5i4.1704 PMC8900652 35310464

[24] McBride O, Butter S, Martinez AP, Shevlin M, Murphy J, Hartman TK, et al. An 18-month follow-up of the Covid-19 psychology research consortium study panel: survey design and fieldwork procedures for Wave 6. Int J Methods Psychiatr Res. 2022;32: e1949. 36217275 10.1002/mpr.1949 PMC9874753

[25] Bzdok D, Altman N, Krzywinski M. Statistics versus machine learning. Nat Methods. 2018;15:233–4. 30100822 10.1038/nmeth.4642 PMC6082636

[26] Stenwig E, Salvi G, Rossi PS, Skjærvold NK. Comparative analysis of explainable machine learning prediction models for hospital mortality. BMC Med Res Methodol. 2022;22:53. 35220950 10.1186/s12874-022-01540-w PMC8882271

[27] Mukumbang FC. Retroductive theorizing: a contribution of critical realism to mixed methods research. J Mix Methods Res. 2023;17:93–114.

[28] Brooks J, McCluskey S, Turley E, King N. The utility of template analysis in qualitative psychology research. Qual Res Psychol. 2015;12:202–22. 27499705 10.1080/14780887.2014.955224 PMC4960514

[29] Power SA, Velez G, Qadafi A, Tennant J. The SAGE model of social psychological research. Perspect Psychol Sci. 2018;13:359–72. 29361241 10.1177/1745691617734863 PMC5946666

[30] Ebrahimi OV, Hoffart A, Johnson SU. Physical distancing and mental health during the COVID-19 pandemic: factors associated with psychological symptoms and adherence to pandemic mitigation strategies. Clin Psychol Sci. 2021;9:489–506.

[31] Magnúsdóttir I, Lovik A, Unnarsdóttir AB, McCartney D, Ask H, Kõiv K, et al. Acute COVID-19 severity and mental health morbidity trajectories in patient populations of six nations: an observational study. Lancet Public Health. 2022;7:e406–16. 35298894 10.1016/S2468-2667(22)00042-1 PMC8920517

[32] Norwegian Institute of Public Health. Folkehelseinstituttet. 2021. Available from: https://www.fhi.no/ss/korona/koronavirus/folkehelserapporten-temautgave-2021/del-1-9/tillit-og-vaksinasjon-i-norge.

[33] Braitman AL, Strowger M, Shipley JL, Ortman J, MacIntyre RI, Bauer EA. Data quality and study compliance among college students across 2 recruitment sources: two study investigation. JMIR Form Res. 2022;6: e39488. 36485020 10.2196/39488 PMC9789498

[34] Spitzer RL, Kroenke K, Williams JBW, Löwe B. A brief measure for assessing generalized anxiety disorder: the GAD-7. Arch Intern Med. 2006;166:1092–7. 16717171 10.1001/archinte.166.10.1092

[35] Pallesen S, Bjorvatn B, Nordhus IH, Sivertsen B, Hjørnevik M, Morin CM. A new scale for measuring insomnia: the bergen insomnia scale. Percept Mot Skills. 2008;107:691–706. 19235401 10.2466/pms.107.3.691-706

[36] Bovin MJ, Marx BP, Weathers FW, Gallagher MW, Rodriguez P, Schnurr PP, et al. Psychometric properties of the PTSD checklist for diagnostic and statistical manual of mental disorders-fifth edition (PCL-5) in veterans. Psychol Assess. 2016;28:1379–91. 26653052 10.1037/pas0000254

[37] Kroenke K, Spitzer RL, Williams JBW. The PHQ-9: validity of a brief depression severity measure. J Gen Intern Med. 2001;16:606–13. 11556941 10.1046/j.1525-1497.2001.016009606.x PMC1495268

[38] Ebrahimi OV, Hoffart A, Johnson SU. Viral mitigation and the COVID-19 pandemic: factors associated with adherence to social distancing protocols and hygienic behaviour. Psychol Health. 2023;38:283–306. 34339328 10.1080/08870446.2021.1960987

[39] Piramuthu S. Input data for decision trees. Expert Syst Appl. 2008;34:1220–6.

[40] Shen Q, Joyce EE, Ebrahimi OV, Didriksen M, Lovik A, Sævarsdóttir KS, et al. COVID-19 illness severity and 2-year prevalence of physical symptoms: an observational study in Iceland, Sweden, Norway and Denmark. Lancet Reg Health – Eur. 2023;35: 100756. 38115966 10.1016/j.lanepe.2023.100756 PMC10730314

[41] Friedman JH. Greedy function approximation: a gradient boosting machine. Ann Stat. 2001;29:1189–232.

[42] Chen T, Guestrin C. XGBoost: a scalable tree boosting system. In: Proceedings of the 22nd ACM SIGKDD International Conference on Knowledge Discovery and Data Mining. New York, NY, USA: Association for Computing Machinery; 2016:785–94. (KDD ’16). 10.1145/2939672.2939785.

[43] Varma S, Simon R. Bias in error estimation when using cross-validation for model selection. BMC Bioinformatics. 2006;7: 91. 16504092 10.1186/1471-2105-7-91 PMC1397873

[44] Dinga R, Penninx BWJH, Veltman DJ, Schmaal L, Marquand AF. Beyond accuracy: measures for assessing machine learning models, pitfalls and guidelines. bioRxiv; 2019. Available from: https://www.biorxiv.org/content/10.1101/743138v1.

[45] Varoquaux G, Colliot O. Evaluating machine learning models and their diagnostic value. In: Colliot O, editor. Machine learning for brain disorders. New York, NY: Springer US; 2023:601–30. 10.1007/978-1-0716-3195-9_20. 37988512

[46] Provost F. Machine learning from imbalanced data sets 101. In Proceedings of the AAAI’2000 workshop on imbalanced data sets 2000 Jul 31 (Vol. 68. AAAI Press; 2000. p. 1-3. https://aaai.org/papers/ws00-05-001-machine-learning-from-imbalanced-data-sets-101/.

[47] Calvert CL, Khoshgoftaar TM. Threshold based optimization of performance metrics with severely imbalanced big security data. 2019 IEEE 31st Int Conf Tools Artif Intell ICTAI. 2019:1328–34.

[48] Leevy JL, Johnson JM, Hancock J, Khoshgoftaar TM. Threshold optimization and random undersampling for imbalanced credit card data. J Big Data. 2023;10:58.

[49] King N. Doing template analysis. In: Qualitative organizational research: core methods and current challenges. 55 City Road: SAGE Publications, Inc.; 2012:426–50. Available from: https://sk.sagepub.com/books/qualitative-organizational-research-core-methods-and-current-challenges/i1774.xml.

[50] Wiltshire G, Ronkainen N. A realist approach to thematic analysis: making sense of qualitative data through experiential, inferential and dispositional themes. J Crit Realism. 2021;20:159–80.

[51] Gale NK, Heath G, Cameron E, Rashid S, Redwood S. Using the framework method for the analysis of qualitative data in multi-disciplinary health research. BMC Med Res Methodol. 2013;13: 117. 24047204 10.1186/1471-2288-13-117 PMC3848812

[52] Steltenpohl CN, Lustick H, Meyer MS, Lee LE, Stegenga SM, Reyes LS, et al. Rethinking transparency and rigor from a qualitative open science perspective. J Trial Error. 2024;4. Available from: https://journal.trialanderror.org/pub/rethinking-transparency/release/1.

[53] Braun V, Clarke V. Conceptual and design thinking for thematic analysis. Qual Psychol. 2022;9:3–26.

[54] Levitt HM, Bamberg M, Creswell JW, Frost DM, Josselson R, Suárez-Orozco C. Journal article reporting standards for qualitative primary, qualitative meta-analytic, and mixed methods research in psychology: the APA Publications and Communications Board task force report. Am Psychol. 2018;73:26–46. 29345485 10.1037/amp0000151

[55] Norwegian Institute of Public Health. Folkehelseinstituttet. Mental illness among adults in Norway. in: Public health report - health status in Norway. 2023. Available from: https://www.fhi.no/he/folkehelserapporten/psykisk-helse/psykiske-lidelser-voksne/.

[56] Steinmetz L. Sociodemographic predictors of and main reasons for COVID-19 vaccine hesitancy in eastern Oslo: a cross-sectional study. BMC Public Health. 2022;22:1878. 36207702 10.1186/s12889-022-14261-y PMC9542469

[57] Poudel AN, Zhu S, Cooper N, Roderick P, Alwan N, Tarrant C, et al. Impact of Covid-19 on health-related quality of life of patients: a structured review. PLoS ONE. 2021;16: e0259164. 34710173 10.1371/journal.pone.0259164 PMC8553121

[58] Tenforde MW. Symptom Duration and Risk Factors for Delayed Return to Usual Health Among Outpatients with COVID-19 in a Multistate Health Care Systems Network — United States, March–June 2020. MMWR Morb Mortal Wkly Rep. 2020;69. Available from: https://www.cdc.gov/mmwr/volumes/69/wr/mm6930e1.htm. 10.15585/mmwr.mm6930e1 PMC7392393 32730238

[59] Zhang H, Li Y, Peng S, Jiang Y, Jin H, Zhang F. The effect of health literacy on COVID-19 vaccine hesitancy among community population in China: the moderating role of stress. Vaccine. 2022;40:4473–8. 35710509 10.1016/j.vaccine.2022.06.015 PMC9174466

[60] Wong JC, Lao CT, Yousif MM, Luga JM. Fast tracking—vaccine safety, efficacy, and lessons learned: a narrative review. Vaccines. 2022;10: 1256. 36016143 10.3390/vaccines10081256 PMC9414382

[61] Phillips R, Gillespie D, Hallingberg B, Evans J, Taiyari K, Torrens-Burton A, et al. Perceived threat of COVID-19, attitudes towards vaccination, and vaccine hesitancy: a prospective longitudinal study in the UK. Br J Health Psychol. 2022;27:1354–81. 35642867 10.1111/bjhp.12606 PMC9347957

[62] Troiano G, Nardi A. Vaccine hesitancy in the era of COVID-19. Public Health. 2021;194:245–51. 33965796 10.1016/j.puhe.2021.02.025 PMC7931735

[63] Andre FE, Booy R, Bock HL, Clemens J, Datta SK, John TJ, et al. Vaccination greatly reduces disease, disability, death and inequity worldwide. Bull World Health Organ. 2008;86:140–6. 18297169 10.2471/BLT.07.040089 PMC2647387

[64] Haas EJ, McLaughlin JM, Khan F, Angulo FJ, Anis E, Lipsitch M, et al. Infections, hospitalisations, and deaths averted via a nationwide vaccination campaign using the Pfizer–BioNTech BNT162b2 mRNA COVID-19 vaccine in Israel: a retrospective surveillance study. Lancet Infect Dis. 2022;22:357–66. 34562375 10.1016/S1473-3099(21)00566-1 PMC8457761

[65] Fieselmann J, Annac K, Erdsiek F, Yilmaz-Aslan Y, Brzoska P. What are the reasons for refusing a COVID-19 vaccine? A qualitative analysis of social media in Germany. BMC Public Health. 2022;22:846. 35484619 10.1186/s12889-022-13265-y PMC9046705

[66] Pertwee E, Simas C, Larson HJ. An epidemic of uncertainty: rumors, conspiracy theories and vaccine hesitancy. Nat Med. 2022;28:456–9. 35273403 10.1038/s41591-022-01728-z

[67] Bavel JJV, Baicker K, Boggio PS, Capraro V, Cichocka A, Cikara M, et al. Using social and behavioural science to support COVID-19 pandemic response. Nat Hum Behav. 2020;4:460–71. 32355299 10.1038/s41562-020-0884-z

[68] Burgess RA, Osborne RH, Yongabi KA, Greenhalgh T, Gurdasani D, Kang G, et al. The COVID-19 vaccines rush: participatory community engagement matters more than ever. The Lancet. 2021;397:8–10. 10.1016/S0140-6736(20)32642-8 PMC7832461 33308484

[69] Ojikutu BO, Stephenson KE, Mayer KH, Emmons KM. Building trust in COVID-19 vaccines and beyond through authentic community investment. Am J Public Health. 2021;111:366–8. 33301352 10.2105/AJPH.2020.306087 PMC7893367

[70] Aw J, Seng JJB, Seah SSY, Low LL. COVID-19 vaccine hesitancy—a scoping review of literature in high-income countries. Vaccines. 2021;9:900. 34452026 10.3390/vaccines9080900 PMC8402587

[71] Freeman D, Loe BS, Yu LM, Freeman J, Chadwick A, Vaccari C, et al. Effects of different types of written vaccination information on COVID-19 vaccine hesitancy in the UK (OCEANS-III): a single-blind, parallel-group, randomised controlled trial. Lancet Public Health. 2021;6:e416–27. 33991482 10.1016/S2468-2667(21)00096-7 PMC8116130

[72] Azarpanah H, Farhadloo M, Vahidov R, Pilote L. Vaccine hesitancy: evidence from an adverse events following immunization database, and the role of cognitive biases. BMC Public Health. 2021;21:1686. 34530804 10.1186/s12889-021-11745-1 PMC8444164

[73] Feitosa ACR, Sampaio LN, Batista AGL, Pinheiro CB. Frequency of fear of needles and impact of a multidisciplinary educational approach towards pregnant women with diabetes. Rev Bras Ginecol E Obstet. 2013;35:111–6. 10.1590/s0100-72032013000300004 23538469

[74] Mackereth P, Hackman E, Tomlinson L, Manifold J, Orrett L. ‘Needle with ease’: rapid stress management techniques. Br J Nurs. 2012;21(Sup14):S18-22. 23252177 10.12968/bjon.2012.21.Sup14.S18

[75] Honora A, Wang KY, Chih WH. How does information overload about COVID-19 vaccines influence individuals’ vaccination intentions? The roles of cyberchondria, perceived risk, and vaccine skepticism. Comput Hum Behav. 2022;130: 107176. 10.1016/j.chb.2021.107176 PMC8730468 35013641

[76] Voils CI, King HA, Maciejewski ML, Allen KD, Yancy WS Jr, Shaffer JA. Approaches for informing optimal dose of behavioral interventions. Ann Behav Med. 2014;48:392–401. 24722964 10.1007/s12160-014-9618-7 PMC4414086

[77] Marhánková JH, Kotherová Z, Numerato D. Navigating vaccine hesitancy: strategies and dynamics in healthcare professional-parent communication. Hum Vaccines Immunother. 2024;20:2361943. 10.1080/21645515.2024.2361943 PMC11168214 38855961

[78] Unfried K, Priebe J. Vaccine hesitancy and trust in sub-Saharan Africa. Sci Rep. 2024;14:10860. 38740790 10.1038/s41598-024-61205-0 PMC11091197

[79] Kola L, Kohrt BA, Hanlon C, Naslund JA, Sikander S, Balaji M, et al. COVID-19 mental health impact and responses in low-income and middle-income countries: reimagining global mental health. Lancet Psychiatry. 2021;8:535–50. 33639109 10.1016/S2215-0366(21)00025-0 PMC9764935

