# Supplementary material for: Modifiable risk factors of vaccine hesitancy: insights from a mixed methods multiple population study combining machine learning and thematic analysis during the COVID-19 pandemic
Source: BMC Med. 2025 Mar 12;23:155. doi: 10.1186/s12916-025-03953-y (PMC11905715; doi:10.1186/s12916-025-03953-y)
Supplement: Supplementary file 1 — Additional File 1: Fig. S1. Role of the key predictors for predicting vaccine hesitancy in the UK sample. (a) The variable importances were calculated based on information gain in the training set. (b) The distribution for each predictor is visualized based on the hold-out test set. All top ten predictors were continuous, ranging from 0 to 100 (Completely disagree to Completely agree for variables 1-2, 6 and 9-10; Not at all to Completely for variables 3-5 and 7; and Not at all threatened to Extremely threatened for variable 8). [file 12916_2025_3953_MOESM1_ESM.pdf]

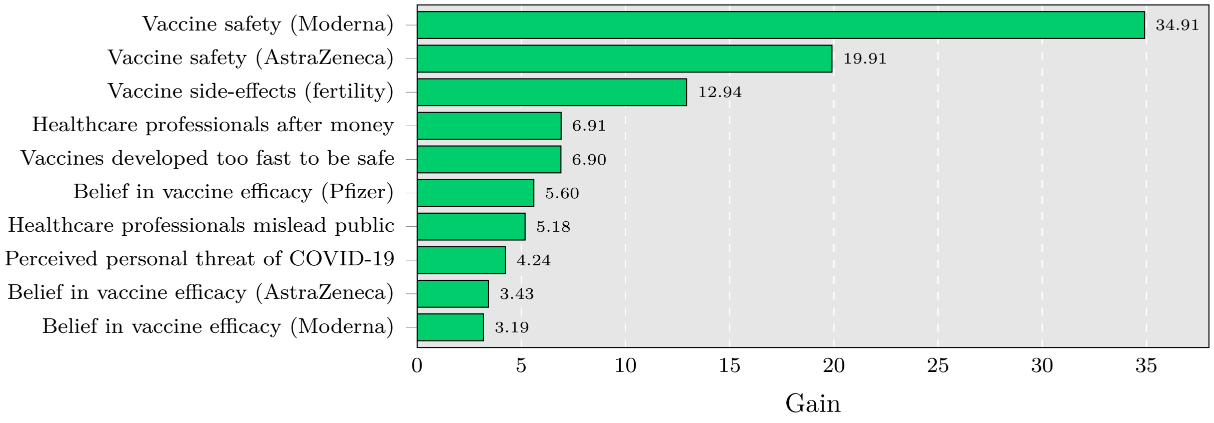

(a) *Ten most important variables and their contribution to the model*

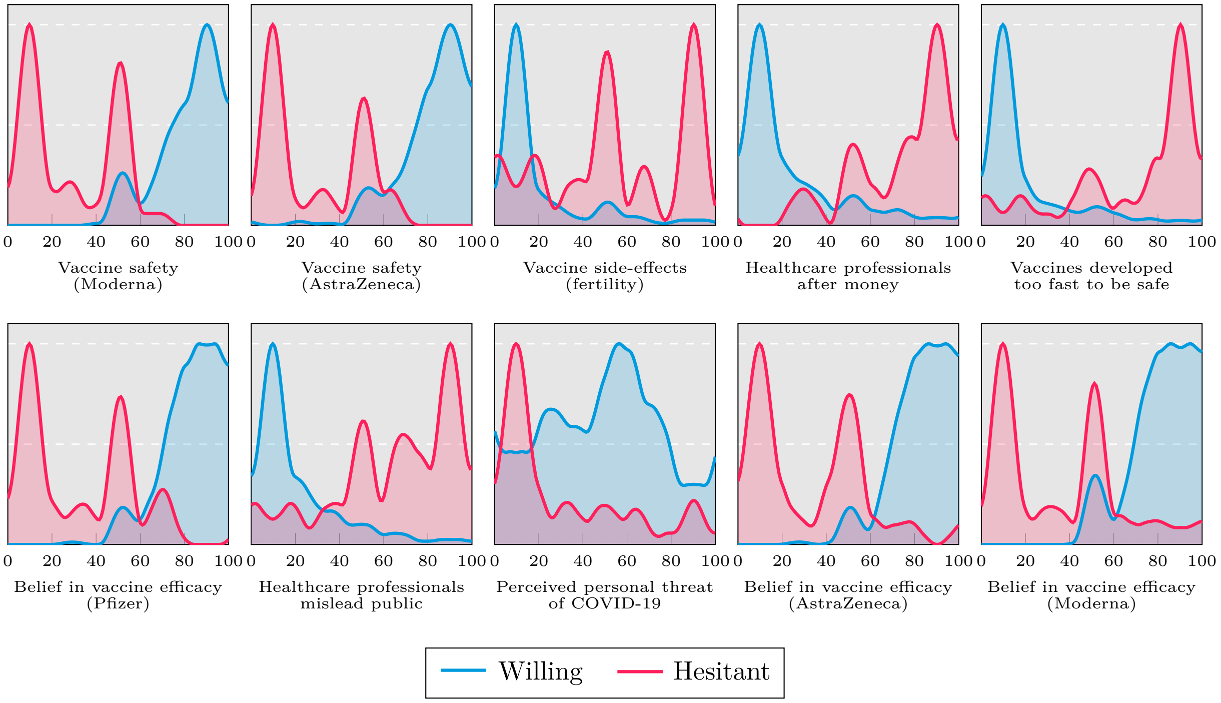

(b) *Distribution of responses for the top ten predictors in the two groups*

Additional File: Figure S1
