# Supplementary material for: Modifiable risk factors of vaccine hesitancy: insights from a mixed methods multiple population study combining machine learning and thematic analysis during the COVID-19 pandemic
Source: BMC Med. 2025 Mar 12;23:155. doi: 10.1186/s12916-025-03953-y (PMC11905715; doi:10.1186/s12916-025-03953-y)
Supplement: Supplementary file 2 — Additional File 2: Table S1. Variables used in the data splitting procedure. Table S2: Hyperparameters and the values tested during tuning. The highlighted values are those that produced the best performing model. Table S3: The variables used in the independent sample replication in the UK dataset along with their corresponding variables in the Norwegian sample. Table S4: Proportion of vaccine hesitant individuals (n = 248 in Norway and n = 109 in UK) within different demographic subgroups. Table S5: Percentage of missingness among the variables with missing values across the vaccine hesitant and vaccine willing subgroups. [file 12916_2025_3953_MOESM2_ESM.pdf]

Additional Tables S1 – S5

Additional Table S1. Variables used in the data splitting procedure.

Variable name	Description	Coding
VaccineHesitancy	Planning to vaccinate against COVID-19 upon availability of a vaccine	Yes; No
Sex	Sex assigned at birth	Females: 0; Males: 1
AgeGroup	Age group	18-30 years; 31-44 years; 45-64 years; and 65 years and above
Education	Highest education to date	Compulsory school: 0; Upper secondary High school: 1; Student: 2; Any university degree: 3
PsychiatricDiagnosisBaseline	Psychiatric diagnosis at baseline	Yes; No
CityResidency	City (versus rural) residency	Yes; No
LivesWithChild	Lives with children (< 18 years)	Yes; No
NotWorking	Unemployed	Yes; No
GovernmentalTrust	I trust in the government and their decision making regarding the pandemic	Completely disagree: 1 to Completely agree: 5
TrustInfoAboutVaccinesHealthOfficials	I trust the information I receive from health officials about vaccines	Completely disagree: 1 to Completely agree: 5

Additional Table S2. Hyperparameters and the values tested during tuning. The highlighted values are those that produced the best performing model.

Parameter	Values
max_depth	{**3**, 6}
learning_rate	{0.01, **0.1**, 0.3}
n_estimators	{100, 250, **500**}
colsample_bytree	{**0.3**, 0.7}
gamma	{0, 1, **10**}

Additional Table S3. The variables used in the independent sample replication in the UK dataset along with their corresponding variables in the Norwegian sample.

Variable name in UK dataset	Description	Coding	Corresponding variable in the Norwegian dataset
COVID19_threat	Perceived personal threat of COVID-19	Not at all threatened: 0 to Extremely threatened: 100	OverconfidenceInC19Avoidance
Vax_conspiracy_4	Vaccines are not safe because they were rapidly developed and tested	Not at all: 0 to Completely: 100	BeliefVaccinesDevelopedToofastToBeSafe
Vax_conspiracy_5	Vaccines can damage fertility	Not at all: 0 to Completely: 100	SideEffectFearsDueToIllness
Trust_Body_1	How much trust do you have in the UK Parliament	Complete trust: 1 to Do not trust at all: 5	GovernmentalTrust
Trust_Body_2	How much trust do you have in the UK Government	Complete trust: 1 to Do not trust at all: 5	GovernmentalTrust
Trust_Body_6	How much trust do you have in your local government (Council or Local authority)	Complete trust: 1 to Do not trust at all: 5	GovernmentalTrust
Science_conspiracy_1	Healthcare professionals and scientists often deceive or mislead the public	Not at all: 0 to Completely: 100	TrustInfoAboutVaccinesHealthOfficials

Science_conspiracy_2	Healthcare professionals and scientists often cover up their mistakes	Not at all: 0 to Completely: 100	TrustInfoAboutVaccinesHealthOfficials
Science_conspiracy_3	Healthcare professionals and scientists are more concerned with making money than taking care of people	Not at all: 0 to Completely: 100	TrustInfoAboutVaccinesHealthOfficials
Science_conspiracy_4	Healthcare professionals and scientists don't know what they are doing	Not at all: 0 to Completely: 100	TrustInfoAboutVaccinesHealthOfficials
Trust_Body_10	How much trust do you have in the scientists	Complete trust: 1 to Do not trust at all: 5	TrustInfoAboutVaccinesHealthOfficials
Trust_Body_11	How much trust do you have in doctors and other health professionals	Complete trust: 1 to Do not trust at all: 5	TrustInfoAboutVaccinesHealthOfficials
Vaccine_beliefs_1	The Pfizer/BioNTech vaccine is effective	Completely disagree: 0 to Completely agree: 100	BeliefVaccinationEfficacyAgainstBeingInfected
Vaccine_beliefs_3	The Oxford/AstraZeneca vaccine is effective	Completely disagree: 0 to Completely agree: 100	BeliefVaccinationEfficacyAgainstBeingInfected
Vaccine_beliefs_5	The Moderna vaccine is effective	Completely disagree: 0 to Completely agree: 100	BeliefVaccinationEfficacyAgainstBeingInfected
Vaccine_beliefs_2	The Pfizer/BioNTech vaccine is safe	Completely disagree: 0 to Completely agree: 100	PerceivedRiskOfVaccinating
Vaccine_beliefs_4	The Oxford/AstraZeneca vaccine is safe	Completely disagree: 0 to Completely agree: 100	PerceivedRiskOfVaccinating
Vaccine_beliefs_6	The Moderna vaccine is safe	Completely disagree: 0 to	PerceivedRiskOfVaccinating

	Completely agree: 100	

Additional Table S4. Proportion of vaccine hesitant individuals (*n* = 248 in Norway and *n* = 109 in UK) within different demographic subgroups.

Variable	Norwegian sample $N_{hesitant}$ of $N_{subgroup}$ (%)	UK sample $N_{hesitant}$ of $N_{subgroup}$ (%)
**Sex**		
Female	205 of 2332 (8.79%)	58 of 419 (13.84%)
Male	43 of 572 (7.52%)	50 of 312 (16.03%)
Missing[a]	0 of 248 (0%)	1 of 109 (0.92%)
**Age (years)**		
18-30	95 of 1131 (8.40%)	23 of 148 (15.54%)
31-44	93 of 907 (10.25 %)	46 of 312 (14.74%)
45-64	49 of 716 (6.84%)	36 of 259 (13.90%)
65+	10 of 153 (6.54%)	4 of 15 (26.67%)
Missing[a]	1 of 248 (0.40%)	0 of 109 (0%)
**University degree**		
Yes	149 of 1767 (8.43%)	25 of 198 (12.63%)
No	98 of 1140 (8.60%)	28 of 142 (19.72%)
Missing[a]	1 of 248 (0.40%)	56 of 109 (51.38%)
**Residency**		
Urban	131 of 1844 (7.10%)	15 of 87 (17.24%)
Rural area	26 of 306 (8.50%)	38 of 253 (15.02%)
Missing[a]	91 of 248 (36.69%)	56 of 109 (51.38%)
**Ethnic status**		
Ethnic majority[b]	235 of 2727 (8.62%)	44 of 292 (15.07%)
Ethnic minority	12 of 179 (6.70%)	9 of 48 (18.75%)
	1 of 248 (0.40%)	56 of 109 (51.38%)
**Psychiatric diagnosis**		
Yes	46 of 526 (8.75%)	23 of 182 (12.64%)
No	201 of 2380 (8.45%)	78 of 517 (15.09%)
Missing[a]	1 of 248 (0.40%)	8 of 109 (7.34%)

Note. [a]Missing responses within the vaccine hesitant group. [b]White British or Irish (UK sample) or white Norwegian (Norwegian sample) ethnicity.

Additional Table S5. Percentage of missingness among the variables with missing values across the vaccine hesitant and vaccine willing subgroups.

Variable name	Total	Willing	Hesitant
Sex	0.72	0.00	0.78
Age	0.67	0.61	0.67
AgeGroup	0.67	0.61	0.67
Education	0.67	0.61	0.67
EmploymentStatus	0.72	0.61	0.73
County	0.72	0.61	0.73
Ethnicity	0.72	0.61	0.73
ImmigrationStatus	0.72	0.61	0.73
Region	0.72	0.61	0.73
CityResidency	26.81	37.58	25.81
LivesWithChild	0.72	0.61	0.73
NotWorking	13.33	22.42	12.49
ParentalStress	70.68	65.45	71.16
SomatoSensoryAmplification	24.14	30.91	23.52
SefEfficacy	26,81	37.58	25.81
IntoleranceOfUncertainty	0.21	0.61	0.17
WhichCloseOthersInfectedC19	64.94	58.79	65.51
CloseOtherHospitalizedC19	64.94	58.79	65.51
CloseOtherICUC19	64.94	58.79	65.51
CloseOtherDeathC19	64.94	58.79	65.51
CloseOthersMaintainedSymptomatologyC19	64.94	58.79	65.51
CloseOtherSymptomLengthC19	64.94	58.79	65.51
Extroversion	16.97	29.09	15.85
Conscienciousness	16.97	29.09	15.85
Neuroticism	16.97	29.09	15.85
Openess	16.97	29.09	15.85
Agreeableness	16.97	29.09	15.85
AutonomyNeeds	26.81	37.58	25.81
AutonomyFrustration	26.81	37.58	25.81
Altruism	26.81	37.58	25.81
ContextualConsideration	26.81	37.58	25.81
PerceivedCompetence	26.81	37.58	25.81
CivilStatus	26.81	37.58	25.81
Mentalization	26.81	37.58	25.81
AvailableSupport	26.81	37.58	25.81
InterpersonalProblems	23.89	31.52	23.18
MediaTypePreference	28.45	40.00	27.38
