# Supplementary material for: Modifiable risk factors of vaccine hesitancy: insights from a mixed methods multiple population study combining machine learning and thematic analysis during the COVID-19 pandemic
Source: BMC Med. 2025 Mar 12;23:155. doi: 10.1186/s12916-025-03953-y (PMC11905715; doi:10.1186/s12916-025-03953-y)
Supplement: Supplementary file 3 — Additional File 3. Document 1. List of variables used in the machine learning model. [file 12916_2025_3953_MOESM3_ESM.pdf]

Additional File: Document 1. List of variables used in the machine learning model

List of the 111 variables used in the machine learning model.

Variable name	Description	Coding
OverconfidenceInC19Avoidance	I do not need to vaccinate as I have managed to avoid the coronavirus so far	Completely disagree: 1 to Completely agree: 5
SideEffectFearsDueToIllness	I am afraid of vaccinating because of possible side-effects or related to underlying disease	Completely disagree: 1 to Completely agree: 5
TrustInfoAboutVaccinesHealthOfficials	I trust the information I receive from health officials about vaccines	Completely disagree: 1 to Completely agree: 5
BeliefVaccinationEfficacyMitigateTransmission	I believe vaccination can help mitigate transmission of infectious disease	Not at all probable: 1 to Highly probable: 5
BeliefInSuperiorityOfNaturalImmunity	It is less risky for me to obtain natural immunity rather than vaccinating against the coronavirus	Completely disagree: 1 to Completely agree: 5
GovernmentalTrust	I trust in the government and their decision making regarding the pandemic	Completely disagree: 1 to Completely agree: 5
BeliefVaccinationEfficacyAgainstBeingInfected	I believe the COVID-19 vaccines are effective in protecting against infection from the coronavirus	Completely disagree: 1 to Completely agree: 5
PerceivedRiskOfVaccinating	How risky or dangerous do you perceive it to vaccinate against the coronavirus?	Not risky at all: 0 to Maximally risky: 100
BeliefVaccinesDevelopedToofastToBeSafe	I believe the vaccines have been developed too fast to be safe.	Completely disagree: 1 to Completely agree: 5
PerceivedDangerOfCOVIDForSociety	Perceived danger of the SARS-CoV-2 virus for society	Not at all serious: 0 to Very serious: 5
Sex	Sex assigned at birth	Females: 0; Males: 1

Age	Age in years	Integer, number of years
AgeGroup	Age group	18-30 years; 31-44 years; 45-64 years; and 65 years and above
Education	Highest education to date	Compulsory school: 0; Upper secondary High school: 1; Student: 2; Any university degree: 3
EmploymentStatus	Employment status	Full time job; Part time; Sick leave; Work clearance allowance; Disability benefits; Retired; Student; Homemaker; Parental leave; Other
County	County of residence	Agder; Innlandet; Møre og Romsdal; Nordland; Oslo; Rogaland; Troms og Finnmark; Trøndelag; Vestfold og Telemark; Vestland; Viken
Ethnicity	Ethnic background	Norwegian; European; Middle-Eastern or North-African; Middle-Asian; East- and South-East Asian; Central and South African; North-American Background; Latin-, Caribbean, and South-American; Oseanic background

ImmigrationStatus	Refugee or immigration status	No; I am a first generation immigrant/refugee; I am a second generation immigrant
Region		East Norway; West Norway; Middle Norway; North Norway
CityResidency	City (versus rural) residency	Yes; No
LivesWithChild	Lives with children (< 18 years)	Yes; No
BMI	Body Mass Index	Numeric
NumberOfChildrenCohabiting	Number of children (< 18 years) participant resides with	Integer, number of children
HealthProfessional	Heath professional (e.g., physician, nurse)	Yes; No
LoneResidency	Lives alone	Yes; No
CivilStatusChange	Civil status change during the pandemic	No; Yes, got in a relationship/civil partnership/married; Yes, became single/divorced; A combination of the above
NotWorking	Unemployed	Yes; No
CurrentPsychiatricTreatment	Receiving current psychiatric treatment	Not seeking any psychological treatment; Treatment related to anxiety; Treatment related to depressive symptoms; Treatment related to loneliness; Treatment related to stress and trauma-related problems; Treatment for loss

		and/or grief; Treatment for obsessive-compulsive problems; and Treatment for other psychological problems
PreviousPsychiatricTreatment	Receiving previous psychiatric treatment	No; For anxiety disorder; Depression; Bipolar or other mood disorder; Eating disorder; Obsessive-compulsive disorder; Trauma and stress disorder; Panic disorder; Insomnia or other sleep disorder; Alcohol or substance use disorder; Personality disorder; Psychotic disorder; Dissociative disorder; Attention-deficit disorder; Treatment for other psychiatric diagnoses
PsychiatricDiagnosisCurrent	Current psychiatric diagnosis	None; Anxiety disorder; Depression; Bipolar or other mood disorder; Eating disorder; Obsessive-compulsive disorder; Trauma and stress disorder; Panic disorder; Insomnia or other sleep disorder; Alcohol or substance use

		disorder; Personality disorder; Psychotic disorder; Dissociative disorder; Attention-deficit disorder; Other psychiatric diagnosis
MedicalComorbidityBeforePandemic	Medical illness from before the pandemic	None; Lung disease; Blood-related disease; Cancer; Heart-related disease; ME/Chronic Fatigue; Congenital disease; "Ear, nose, or throat (otolaryngology) disease; Eye disease; Infectious disease; Immune or inflammatory disease; Metabolic or endocrine-related disease; Musculoskeletal and joint disease; Neurological disease
MedicalComorbidityCurrent	Current medical illness	None; Lung disease; Blood-related disease; Cancer; Heart-related disease; ME/Chronic Fatigue; Congenital disease; "Ear, nose, or throat (otolaryngology) disease; Eye disease; Infectious disease; Immune or inflammatory disease; Metabolic or endocrine-related disease; Musculoskeletal

		and joint disease; Neurological disease
ChronicDiseaseCurrent	Current chronic illness	Yes; No
LearningDifficultiesCurrent	Learning difficulties	None; Dyslexia; Dysgraphia or dysorthographia; dyscalculia; General learning difficulties; Other learning difficulties
DepressionLevel	Patient Health Questionnaire-9 (PHQ-9), total score	Numeric, range: 0-29
AnxietyLevel	Generalized Anxiety Disorder-7 (GAD-7), total score	Numeric, range: 0-21
DepressionFunctioning	Depressive symptoms interfering with daily life functioning	Not at all: 0 to Nearly every day: 3
AnxietyFunctioning	Anxiety symptoms interfering with daily life functioning	Not at all: 0 to Nearly every day: 3
Loneliness	UCLA Loneliness Scale (ULS-8)	Numeric, range: 8-32
ObsessiveThoughtsC19	Obsession with COVID-19 Scale (OCS)	Numeric, range: 0-16
SleepDifficulties	Bergen Insomnia Scale (BIS)	Numeric, range: 0-42
WellBeing	Short Warwick-Edinburgh Mental Wellbeing Scale (SWEMWBS)	Numeric, range: 7-35
TraumaSymptoms	PTSD Checklist for DSM-5 (PCL-5)	Numeric, range: 0-80
ParentalStress	Danish Parental Stress Scale (PSS) – Short Form	Numeric, range: 3-15
SomatoSensoryAmplification	SomatoSensory Amplification Scale (SSAS) – Short Form	Numeric, range: 4-20
SelfEfficacy	General Self-Efficacy Scale (GSE) – Short Form	Numeric, range: 2-8

Fatigue	The Shortened Fatigue Questionnaire (SFQ)	Numeric, range: 4-28
SomaticSymptoms	Patient Health Questionnaire-15 (PHQ-15)	Numeric, range: 0-30
C19Infection	Infected by SARS-CoV-2	Yes; No
BedriddenC19Infection	Number of days bedridden during SARS-CoV-2 infection	Numeric, range: 0-30
VaccineAccessibility	Experience of vaccines being easy to access	Strongly Disagree: 1 to Strongly Agree: 5
BeliefinEfficacyOfMitigationProtocols	Social distancing protocols are effective in mitigating transmissions	Highly unlikely/Not at all: 0 to Highly likely: 5
PerceivedDangerOfCOVIDForSelf	Perceived danger of being infected by SARS-CoV-2	Not at all serious: 0 to Very serious: 5
PerceivedDangerOfCOVIDForOthers	Perceived danger of others being infected by SARS-CoV-2	Not at all serious: 0 to Very serious: 5
PerceivedDangerC19Overall	Perceived danger of the SARS-CoV-2 in general	Numeric, range: 0-15
AdherenceHygienicProtocols	Adherence to hygienic behavior recommendations	Numeric, range: 0-28
AdherenceSDPProtocols	Adherence to social distancing protocols	Numeric, range: 0-20
QuarantineExposureFrequency	Number of times quarantined	Integer
IsolationExposureFrequency	Number of times in isolation	Integer
PhysicalSocialContact	Extent of engagement in physical social contact during past two weeks	Not at all: 0 to Extremely: 5
DigitalSocialContact	Extent of engagement in digital social contact during past two weeks	Not at all: 0 to Extremely: 5
NotLeftOwnHomePast14Days	Number of days subject has not been outside their home during the past two weeks	Integer, range: 0-14

PhysicalActivity	Physical activity frequency during the past two weeks	Integer, range: 0-14
InformationNewspapers	Frequency of information acquisition from newspapers during the last month	Not at all: 0 to 7: Multiple times per hour
InformationTV	Frequency of information acquisition from television during the last month	Not at all: 0 to 7: Multiple times per hour
InformationSocialMedia	Frequency of information acquisition from social media during the last month	Not at all: 0 to 7: Multiple times per hour
InformationsForumsBlogs	Frequency of information acquisition from forums and/or blogs during the last month	Not at all: 0 to 7: Multiple times per hour
InformationPeers	Frequency of information acquisition from peers during the last month	Not at all: 0 to 7: Multiple times per hour
InformationOther	Frequency of information acquisition from other sources during the last month	Not at all: 0 to 7: Multiple times per hour
InformationActiveAvoidance	Frequency of active decision to avoid all information/news sources during the last month	Not at all: 0 to 7: Multiple times per hour
TotalInformationObtainmentTime	Mean score of information acquisition behavior across sources	Numeric, range: 0-7
FinancialDifficulties	Financial difficulties	Not at all: 0 to Very much: 4
FinancialOccupationalWorry	Extent of worry about losing job or personal economy	Not at all: 0 to Nearly every day: 3
FearOwnHealthC19	Fear of own health from SARS-CoV-2	Numeric, range: 0-6
FearSignificantOthersDyingC19	Worry about significant others dying from SARS-CoV-2	Not at all: 0 to Nearly every day: 3
FearOthersHealthC19	Worry about others’ health related to SARS-CoV-2	Numeric, range: 0-6

FearOfTransmittingC19Others	Worrying about transmitting others with SARS-CoV-2	Not at all: 0 to Nearly every day: 3
HealthAnxietyAboutC19	Health anxiety related to SARS-CoV-2	Numeric, range: 0-6
FearDeath	Fear of dying from SARS-CoV-2	Not at all: 0 to Nearly every day: 3
IncreaseTobaccoUse	Increased usage of tobacco during the pandemic	No increase: 0 to Much more than before the pandemic: 3
IncreaseAlcConsumption	Increased consumption of alcohol during the pandemic	No increase: 0 to Much more than before the pandemic: 3
IncreaseFoodIntake	Increased food consumption during the pandemic	No increase: 0 to Much more than before the pandemic: 3
IncreaseSweetsConsumption	Increased sweets consumption during the pandemic	No increase: 0 to Much more than before the pandemic: 3
EmotionRegulationDifficulties	Difficulties in Emotion Regulation Scale (DERS) – Short Form	Numeric, range: 6-30
NegativeMetacognitions	Cognitive-attentional Syndrome Questionnaire (CAS-1) – Negative metacognitions subscale	Numeric, range: 0-400
MaladaptiveCopingStrategies	Cognitive-attentional Syndrome Questionnaire (CAS-1) – Maladaptive coping strategies subscale	Numeric, range: 0-64
IntoleranceOfUncertainty	Intolerance of Uncertainty Scale (IUS) – Short Form	Numeric, range: 3-15
ExecutiveFunctioning	Self-Report Measure of Executive Function for Administration via the Internet (WebEXEC)	Numeric, range: 1-24
Impulsivity	Impulsive behavior	No problem with this at all: 0 to Frequent problems with this: 4

CloseOthersInfectedC19	Significant other infected by SARS-CoV-2	Yes; No
WhichCloseOthersInfectedC19	Which significant other infected by SARS-CoV-2	Partner; Child; Parent; Sibling; Grandparent; Close Friend; Grandchild; Other family; Other
CloseOtherHospitalizedC19	Significant other hospitalized after SARS-CoV-2 infection	Yes; No
CloseOtherICUC19	Significant other sent to ICU after SARS-CoV-2 infection	Yes; No
CloseOtherDeathC19	Significant other deceased from SARS-CoV-2 infection	Yes; No
CloseOthersMaintainedSymptomatologyC19	Significant other suffers from sustained symptoms after COVID-19 infection	Yes; No
CloseOtherSymptomLengthC19	Length suffering from long-term symptom experience after SARS-CoV-2 infection by significant other	More than 1 month: 1; 1-3 months: 2; 4-6 months: 3; More than 6 months: 6
Extroversion	Brief Version of the Big Five Personality Inventory (BFI-10) – Extroversion subscale	Numeric, range: 2-10
Conscientiousness	Brief Version of the Big Five Personality Inventory (BFI-10) – Conscientiousness subscale	Numeric, range: 2-10
Neuroticism	Brief Version of the Big Five Personality Inventory (BFI-10) – Neuroticism subscale	Numeric, range: 2-10
Openness	Brief Version of the Big Five Personality Inventory (BFI-10) – Openness subscale	Numeric, range: 2-10
Agreeableness	Brief Version of the Big Five Personality Inventory (BFI-10) – Agreeableness subscale	Numeric, range: 2-10
AutonomyNeeds	Experience a need for autonomy	Not at all: 0 to Nearly every day: 3
AutonomyFrustration	Experience autonomy need deficit and frustration	Not at all: 0 to Nearly every day: 3

Altruism	Desire to help society and societal peers	Not at all: 0 to Nearly every day: 3
ContextualConsideration	Often think about the health and welfare of others around me	Not at all: 0 to Nearly every day: 3
PerceivedCompetence	Belief in own ability to deal with novel crises	Not at all: 0 to Nearly every day: 3
CivilStatus	Married or in a civil partnership	Yes; No
Mentalization	Others describing me as good of perceiving of others internal states	Does not apply to me at all: 1 to Applies to me nearly all the time: 5
AvailableSupport	Experience sufficient social support by others	Not at all: 0 to Nearly every day: 3
InterpersonalProblems	Inventory of Interpersonal Problems – Short Form	Numeric, range: 0 to 68
MediaTypePreference	Preferred media platform type	Recognized and source-checked national, regional and local platforms; Unmonitored information platforms
