# Supplementary material for: Modifiable risk factors of vaccine hesitancy: insights from a mixed methods multiple population study combining machine learning and thematic analysis during the COVID-19 pandemic
Source: BMC Med. 2025 Mar 12;23:155. doi: 10.1186/s12916-025-03953-y (PMC11905715; doi:10.1186/s12916-025-03953-y)
Supplement: Supplementary file 4 — Additional File 4. Document 2. Audit trail of the analytic procedure in the thematic analysis. [file 12916_2025_3953_MOESM4_ESM.pdf]

Additional File: Document 2. Audit trail of the analytic procedure in the thematic analysis

To analyze the 52 open-ended survey responses from the Norwegian adults who had reported that they had not taken and will not take the COVID-19 vaccines, the second and third authors of the study used the template analysis approach to thematic analysis (1,2). This method was chosen because it involves a high degree of structure in the analysis of data while retaining flexibility by creating a coding template while working with the data. This template is then revised and refined as the analysis develops. The following supplementary document presents an audit trail of our analytical procedure to increase transparency (3,4). As per King (2) and Brooks et al. (1), our approach is divided into six steps.

1. Become familiar with the accounts to be analyzed

In the first step, both authors familiarized themselves with the data by reading and re-reading the dataset consisting of the 53 open-ended responses provided by those participants who had chosen to not get vaccinated to the question: "If you did not get vaccinated against COVID-19, could you please explain what contributed to you not wanting to get vaccinated?". One of the responses was excluded from further analysis due to the response merely stating: "No", thus, not providing any underlying reason for the unwillingness to vaccinate, leaving 52 responses. The full dataset cannot be publicly shared due to confidentiality.

2. Carry out preliminary coding of the data

The second step involves the first coding of the data that eventually results in the initial coding template (1). Coding is done by highlighting anything in the text that can contribute to the understanding of the research question, which was: "What reasons do the participants provide for their choice of not wanting to get vaccinated?". Both authors independently coded the full dataset line-by-line in two columns: one for manifest meaning that was close to the participants' words, and one for potential latent meaning that allowed for the researchers' interpretation of the meanings in the dataset. Below is an example from one of the researchers' first coding of the data (Table S6):

Supplementary Table S6. Example from initial coding.

Respondents' answer	Initial codes (Manifest meaning)	Initial codes (Latent meaning)
I am young and healthy. I have little contact with older or vulnerable people. The vaccine provides temporary protection. COVID-19 is here to stay. Given the known side-effects, I believe it has been unethical to include children and young people in the vaccination campaign, especially in the way it has been done. Newspapers have recently published a lot of hatred and misleading information about the unvaccinated, among other things. My lack of vaccination is therefore partly a personal assessment of the personal health risk posed by the choices presented to me, and a protest against some of the discussed political points.	Young and healthy<br>Limited contact with high-risk groups<br>Vaccine provides only temporary protection<br>COVID-19 here to stay<br>Unethical to vaccinate children due to side-effects<br>Media spreads hatred and misinformation about the unvaccinated<br>Assessment of my own health risk as low<br>A protest against the political points	Unnecessary if not in a high-risk group<br>Unnecessary if no contact with high-risk groups<br>Lack of belief in the necessity of the vaccine<br>Political disagreement with the campaigns<br>Dissatisfaction with the media's portrayal<br>Non-vaccination politicized (i.e., as a protest)<br>Autonomy - an active choice

([Gender anonymized], [years anonymized])

3. Organize the emerging themes into meaningful clusters

After both authors independently coded the whole dataset for manifest and latent meaning, they jointly went through both fully coded versions. From this process, the authors clustered meaningful codes into hierarchical themes in the coding template, focusing on the research question of what reasons the participants provided for their choice of not wanting to get vaccinated, with the aim of obtaining a full mapping of the different reasons provided. Some participants gave single reasons, while others provided various reasons. When giving various reasons, all were coded, as illustrated in the above example (Table S6) of coding from the same participant.

4. Define an initial coding template

The clustering of codes from stage 3 in the analytical process resulted in the initial coding template that is shown in Table S7. Here we highlight that we thus slightly deviated from the standard approach in Template analysis, where you typically make a coding template from a few of the interviews. This has been pointed out as a potential weak spot of template analysis, as one could risk overlooking new aspects in the latter interviews (5). We thus chose to do a thorough initial coding of all the material first, to avoid this limitation, and subsequently build our coding template.

Supplementary Table S7. Initial coding template.

**Superordinate codes (main theme level)**	**Subordinate code (sub-theme level)**	**Codes at sub-sub level (sub-sub theme level)**
General reasons (non-related to COVID-19	Fear of needles<br>Fear related to own underlying disease<br>Skeptical to vaccines in general	

vaccines or COVID-19)		
Experience-based health-related reasons	Negative experiences with previous vaccinations	
	Fear that would not get healthcare follow up if needed based on previous experiences	
Side-effects of COVID-19 vaccines	Long-term side-effects not clear	
	Scared of concrete side-effects	Menstruation changes
		Personally know others who have had severe side-effects
		More harmful side-effects than previous debated vaccines (such as the swine flu vaccine)
		Fertility reasons
	The risk of side-effects perceived as greater than risk associated with COVID-19	Some on the fence: Might take vaccine if this changes
Aspects with COVID-19	Not so dangerous	I am not specifically in the risk group
		I am not in the risk of infecting others
		Themselves/acquaintances have only had mild symptoms of COVID-19
	Serves from human activity on nature, nature’s way of “taking back”/”re-balancing”	
Distrust	Lack of trust, authorities	
	Lack of trust, pharmaceutical industry	

		Do not want to support pharmaceutical industry
	Mainstream media misleading people	
		"Uncensored" media telling the truth
	Censorship of alternative voices	
Alternatives to the vaccine	Avoiding crowds	
	The course of nature	Acceptance of own death as course of nature
		Natural for old people to pass away
		Nature's way of handling overpopulation
	Natural immunity	Natural herd immunity
	Avoiding taking a stance because overwhelming	
	Own control: Strengthen own immune system, taking vitamin supplements, exercise, control diet	
General about the nature of the vaccine	The vaccine should be given to those who want/need it more	
	Process too quick, a lot of uncertainty	
	Not offering much protection	Does not hinder the spread of COVID-19
		Alarming to include kids and youth
	Disqualifying these as real vaccines	
	Not interested in the repeated rounds of vaccinations that are required	

Portrayal of the unvaccinated	Pressure to get vaccinated<br>Unwilling due to the portrayal of the unvaccinated

5. Apply the initial template to data and modify as necessary

In this phase, both authors independently applied the initial coding template to the entire dataset, while considering whether any of the themes defined in this template could represent each data extract. After this the authors investigated the degree of overlap in their use of codes from the template. Of 317 coding instances 282 instances overlapped at the main theme level (88.96%) and 260 at the sub-theme level (82.02%). In the instances where the authors had coded differently from the template or had discovered data instances where the template did not fit, codes were discussed, and the coding template revised. For example, at one instance one of the authors coded an extract under the superordinate level code "Aspects with COVID", while the other author coded the same under the subordinate code "Risk of side-effects greater than the risk of COVID", demonstrating that these codes needed refinement and clarification. As emphasized by Brooks et al. (1), the initial template should be revised in an iterative process until a rich and comprehensive representation of the interpretation of the data is achieved.

6. Finalize the template and apply it to the full dataset

The authors finalized the coding template and applied it to the full dataset. This template is presented as the six themes that are presented in the article. Below (Table S8) is the final coding template consisting of all levels of final codes. This final coding template is an extended thematic map of the same version as the one presented in the paper, but including all sub-levels.

Supplementary Table S8: Extended thematic map developed from coding template.

Theme	Subtheme	*N*	Sub-subtheme	***n*** **= Total respondents providing this as a reason**<br>**# = Respondent number**
Theme 1: Unnecessary Vaccines<br>(*n*=17)	1.1 COVID-19 in general not dangerous	*n*=12	1.1.1 Not at all dangerous	*n*=3<br>(#19, #35, #39)
			1.1.2 Not as dangerous as it is portrayed to be	*n*=7<br>(#4, #10, #17, #33, #41, #47, #48)
			1.1.3 Participants/acquaintances have only had mild symptoms of COVID-19	*n*=3<br>(#10, #23, #51)
	1.2 Perceiving oneself as not personally being at risk	*n*=9		*n*=9<br>(#4, #7, #9, #10, #14, #23, #24, #42, #48)
Theme 2: Inefficient Vaccines<br>(*n*=19)	2.1 Does not offer much protection	*n*=11	2.1.1 Does not have a good effect	*n*=5<br>(#1, #22, #23, #38, #47)
			2.1.2 Effect does not last	

				n=6<br>(#4, #7, #9, #20, #27, #40)
	2.2 Does not hinder transmission	*n*=7		*n*=7<br>(#4, #10, #11, #13, #19, #40, #42)
	2.3 Disqualifying these as *real* vaccines	*n*=4		*n*=4<br>(#5, #13, #29, #52)
Theme 3: Frightening Vaccines (*n*=32)	3.1 Fear of side-effects	*n*=26	3.1.1 Generally fearing side-effects of the vaccines	*n*=12<br>(#4, #5, #7, #13, #14, #20, #24, #27, #35, #38, #45, #49)
			3.1.2 Uncertainty about possible long-term side-effects	*n*=7<br>(#11, #12, #27, #28, #36, #37, #50)
			3.1.3 Perceiving risks of side-effects as larger than risks of infection with COVID-19	*n*=6<br>(#10, #19, #23, #38, #48, #51)

			3.1.4 Personally know others who have experienced side-effects	*n*=4<br>(#10, #29, #38, #51)
			3.1.5 Worry about menstruation changes	*n*=4<br>(#21, #48, #50, #51)
			3.1.6 Pregnancy/fertility reasons for fear of side-effects	*n*=3<br>(#23, #32, #48)
	3.2 Fear of not getting healthcare follow-up if needed	*n*=3		*n*=3<br>(#20, #21, #43)
	3.3 Vaccine approvals too quick	*n*=8		*n*=8<br>(#4, #9, #13, #16, #17, #25, #27, #40)
Theme 4: Distrust and Polarization<br>(*n*=18)	4.1 Authorities' vaccine handling	*n*=10	4.1.1 Not providing sufficient and/or correct information	*n*=7<br>(#13, #14, #16, #18, #19, #31, #40)
			4.1.2 Distrusting authorities' vaccine handling	*n*=7<br>(#3, #4, #16, #18, #31, #40, #52)

4.2 Pharmaceutical industry	*n*=3	4.2.1 Do not want to support pharmaceutical industry economically	*n*=1 (#38)
		4.2.2 Distrusting pharmaceutical industry	*n*=3 (#3, #16, #38)
4.3 Mainstream media's vaccine presentation	*n*=7	4.3.1 Mainstream media as misleading	*n*=3 (#7, #31, #35)
		4.3.2 Refers to vaccine critical sources	*n*=6 (#5, #19, #25, #31, #35, #48)
4.4 Portrayal of the unvaccinated	*n*=6	4.4.1 Polarizing portrayals contributing to unwillingness to get vaccinated	*n*=3 (#7, #20, #40)
		4.4.2 Pressure to get vaccinated	*n*=3 (#19, #22, #31)

Theme 5: Alternatives to Getting Vaccinated (*n*=15)	5.1 Natural immunity	*n*=4	5.1.1 Natural herd immunity as the solution to the pandemic	*n*=1 (#4)
			5.1.2 Own natural immunity through infection with COVID-19 gives better protection than the vaccine	*n*=3 (#9, #30, #42)
	5.2 The course of nature	*n*=5	5.2.1 Viewing own potential death as natural	*n*=3 (#8, #15, #39)
			5.2.2 Natural for old people to pass away	*n*=1 (#14)
			5.2.3 Pandemic as nature's way of handling overpopulation/misuse	*n*=2 (#8, #9)
	5.3 Own health measures	*n*=2	5.3.1 Strengthen own immune system	*n*=1 (#40)
			5.3.2 Staying physically fit	*n*=1 (#48)
	5.4 Avoid infections	*n*=3	5.4.1 Avoid places where one can get infected	*n*=2 (#24, #44)

			5.4.2 Avoid people in vulnerable groups	*n*=1 (#7)
	5.5 Avoidance as easier than taking a stance	*n*=2		*n*=2 (#2, #20)
Theme 6: Reasons Related to Vaccines in General (*n*=12)	6.1 Fear of needles	*n*=4		*n*=4 (#6, #20, #38, #46)
	6.2 Fear related to own underlying disease	*n*=4		*n*=4 (#21, #31, #34, #45)
	6.3 Negative experiences with previous vaccination/ medication	*n*=4		*n*=4 (#26, #29, #33, #50)

References

1. Brooks J, McCluskey S, Turley E, King N. The Utility of Template Analysis in Qualitative Psychology Research. Qual Res Psychol. 2015 Apr 3;12(2):202–22.

2. King N. Doing template analysis. In: Qualitative Organizational Research: Core Methods and Current Challenges [Internet]. 55 City Road: SAGE Publications, Inc.; 2012 [cited 2024 Jul 23]. p. 426–50. Available from: https://sk.sagepub.com/books/qualitative-organizational-research-core-methods-and-current-challenges/i1774.xml

3. Levitt HM, Bamberg M, Creswell JW, Frost DM, Josselson R, Suárez-Orozco C. Journal article reporting standards for qualitative primary, qualitative meta-analytic, and mixed methods research in psychology: The APA Publications and Communications Board task force report. Am Psychol. 2018 Jan;73(1):26–46.

4. Steltenpohl CN, Lustick H, Meyer MS, Lee LE, Stegenga SM, Reyes LS, et al. Rethinking Transparency and Rigor from a Qualitative Open Science Perspective. J Trial Error [Internet]. 2024 May 24 [cited 2024 Jul 23];4(1). Available from: https://journal.trialanderror.org/pub/rethinking-transparency/release/1

5. Braun V, Clarke V. Conceptual and design thinking for thematic analysis. Qual Psychol. 2022;9(1):3–26.
